# Integrating Consensus-Based Group Decision Making into the Graph Model for Conflict Resolution in Complex Conflict Environments

**DOI:** 10.3390/e28060636

**Published:** 2026-06-04

**Authors:** Hengjie Zhang, Xiaoying Lu, Fang Wang

**Affiliations:** Business School, Hohai University, Nanjing 211100, China; hengjiezhang@hhu.edu.cn (H.Z.); xiaoyinglu@hhu.edu.cn (X.L.)

**Keywords:** graph model for conflict resolution, consensus-based group decision making, option prioritization, fairness concern, optimization model

## Abstract

Complex conflict environments often involve composite conflicting parties composed of multiple individuals with divergent assessments, posing significant challenges for preference elicitation and conflict analysis in the Graph Model for Conflict Resolution (GMCR). Existing option prioritization methods in GMCR rely on ordinal rankings of statement importance and binary evaluations of state support, which limits their ability to capture nuanced and heterogeneous preferences. Moreover, current GMCR frameworks lack a systematic mechanism to reconcile divergent individual assessments and construct collective preferences. To address these gaps, this study integrates consensus-based group decision making into the GMCR framework through flexible assessments. Specifically, pairwise comparisons are employed to represent the relative importance of statements, while continuous values in [0, 1] are used to characterize the degree of statement support for states. To reconcile heterogeneous assessments, a minimum adjustment-based consensus reaching process is developed. Furthermore, grounded in inequity aversion theory from behavioral economics, fairness concern is incorporated to model individuals’ sensitivity to adjustment differences between themselves and others. Based on this mechanism, two minimum adjustment consensus models with fairness concern are proposed. The resulting consensus-based preferences are subsequently integrated into GMCR stability analysis to identify equilibrium solutions. A supply chain carbon reduction case is used to illustrate the implementation process and applicability of the proposed framework.

## 1. Introduction

Conflicts naturally emerge when multiple conflicting parties, guided by distinct value systems, simultaneously pursue their own objectives and interests through strategic interactions [1,2]. A range of methods have been developed for analyzing and resolving conflicts, including game theory [3], metagame analysis [4], conflict analysis [5], and graph models for conflict resolution (GMCR) [6]. Among these, GMCR stands out by integrating both qualitative and quantitative methods, making full use of relative preference information without requiring complete utility functions or extensive datasets [7]. It provides a flexible and systematic framework for modeling, analyzing, and managing real-world conflicts [8]. Consequently, GMCR has been widely applied across diverse domains [9,10,11,12,13,14].

The GMCR consists of two fundamental components [6,15]: **(1) Conflict modeling:** this process involves identifying each conflicting party’s options within a given conflict, generating feasible states from these options, constructing a state transition diagram to model interactions among conflicting parties, and eliciting the preferences [10,16]. **(2) Stability analysis:** this process evaluates the equilibrium properties of states by examining whether any conflicting party has an incentive to unilaterally deviate under specified stability definitions [7], including Nash stability [17], general metarationality (GMR) [5], symmetric metarationality (SMR) [5], and sequential stability (SEQ) [18].

Preference information plays a critical role in GMCR because it directly affects the strategic decisions of conflicting parties and the formation of equilibria. To elicit conflicting parties’ preferences, three primary methods have been developed: direct ranking, option weighting, and option prioritization [19]. In the direct ranking method, preferences are derived from pairwise comparisons of states [7], while the option weighting method assigns importance weights to each option [19]. In contrast, the option prioritization approach constructs preferences using two key components: the relative importance of statements and binary assessments (T or F) that indicate whether each statement holds in a given state [10]. Building on this foundational method, numerous extensions have been proposed to better capture preference information [10,20,21,22]. Despite the valuable insights provided by existing research on the GMCR, preference elicitation and conflict analysis in complex conflict environments still face two critical challenges:

(1) Coarse informational granularity limits the capture of nuanced preferences. In conventional option prioritization, statement importance is predominantly evaluated through simple ordinal rankings, while state support relies strictly on binary evaluations (T or F). However, this rigid informational granularity fails to capture nuanced variations in relative statement importance or the continuous degrees of state support. Consequently, existing methodologies lack the flexible assessments required to accurately depict heterogeneous preference information in intricate real-world dispute resolutions.
(2) Absence of a systematic mechanism to reconcile internal divergences within conflicting parties. In complex real-world conflicts, a conflicting party is often not a homogeneous entity but rather groups composed of multiple individuals, referred to as a composite conflicting party (CCP) [20]. Due to divergent value systems and knowledge backgrounds, individuals within a CCP frequently exhibit internal divergences in their assessments. While recent studies have started to acknowledge the role of CCPs in conflict resolution [7,12], current GMCR frameworks still lack an effective consensus-based group decision making (GDM) mechanism to reconcile these heterogeneous individual assessments and logically construct collective preferences.

To address these limitations, the study integrates consensus-based GDM into the GMCR framework to effectively manage conflicts and assessment divergences in complex conflict environments. In real-world conflicts, a conflicting party may consist of multiple individuals; however, the conflicting party as a whole must ultimately take unified strategic actions. Therefore, this study reconciles divergent individual assessments, derives collective preferences over feasible states, and incorporates these preferences into GMCR stability analysis to identify possible equilibrium solutions. The main contributions of this study are summarized as follows:

(1) To overcome the limited granularity of option prioritization in the GMCR, this study introduces flexible assessment for preference elicitation in complex conflict environments. Specifically, pairwise comparison is used to represent the relative importance of statements, while continuous values within the interval [0, 1] are utilized to characterize the degree of statement support for states. By integrating these two types of assessment information, the preference values of feasible states can be obtained for subsequent GMCR stability analysis. This refined representation enables a more precise and expressive modeling of heterogeneous assessments.
(2) To reconcile divergent individual assessments within CCPs, this study integrates consensus-based GDM into the GMCR framework. Considering that individuals are sensitive to adjustment differences between themselves and others during the consensus reaching process (CRP), fairness concern is incorporated into the framework. Accordingly, two minimum adjustment consensus models with fairness concern are developed. Consequently, consensus-based preferences across all CCPs are derived and enable equilibrium identification through stability analysis.
(3) A supply chain carbon reduction conflict case is presented to illustrate the implementation process of the proposed framework. The case shows how heterogeneous assessments within CCPs can be coordinated, how consensus-based preferences can be generated, and how such preferences can be incorporated into GMCR stability analysis.

The remainder of this study is summarized as follows. Section 2 reviews the related literature and provides the basic knowledge required for this study, including GMCR, CRP, and pairwise comparison relations. Next, Section 3 describes the complex conflict problem and proposes the corresponding resolution framework. Section 4 introduces an integrated framework that incorporates CRP into GMCR in detail. The implementation process of the proposed framework is illustrated through a supply chain carbon emission reduction case in Section 5. Finally, Section 6 concludes this study and outlines promising directions for future research.

## 2. Literature Review and Basic Knowledge

This section provides a brief review of the related literature and introduces the necessary background on GMCR, CRP, and pairwise comparison relations.

### 2.1. Literature Review

**(1)**  **Graph model for conflict resolution**

GMCR is an insightful methodology for conflict analysis and resolution [7,15]. Since its proposal by Kilgour et al. (1987) [16] and Fang et al. (1993) [6], GMCR has been widely applied to a broad range of practical conflicts due to its simplicity and flexibility, including environmental disputes [9,10,11], water conflicts [12,13], and business negotiations [14].

Preference information plays a fundamental role in GMCR because it directly affects the evolution of a conflict and the formation of equilibria. Therefore, the modeling and elicitation of preference information have long been central issues in GMCR research. For preference modeling, most previous studies were within the framework of crisp preferences [7]. Subsequently, to address preference uncertainty in conflicts, a series of preference structures have been introduced into GMCR, such as strength of preferences [23], grey preferences [24], intuitionistic preferences [25], probabilistic preferences [26,27], and linguistic preferences [14]. For eliciting the preferences, three primary methods have been developed within the GMCR framework: direct ranking, option weighting, and option prioritization [19]. Among them, option prioritization has been widely used as an effective method for eliciting preferences because it does not require conflicting parties to directly compare all feasible states. Instead, conflicting parties express their preference information through a set of prioritized statements, which makes the elicitation process more structured and interpretable. Building on the foundational option prioritization method, researchers have proposed several extensions to better capture preference information. For example, Bashar et al. [28] developed an extended option prioritization method by using numerical values within the interval [0, 1] to represent the possibility of option selection; Chen et al. [10] refined option prioritization by incorporating three-way decision theory in GMCR; Li et al. [21] extended option prioritization in GMCR using a general grey number to represent the uncertainty of statements; and Yu et al. [22] extended the option prioritization method to express the hybrid preferences of conflicting parties.

Most existing GMCR studies treat each conflicting party as an individual. To investigate conflict situations in which each conflicting party consists of multiple individuals, Wu et al. [20] proposed the concept of CCPs within the GMCR framework. Subsequently, some studies have begun to examine conflict situations involving CCPs. For example, Wu et al. [13] incorporated the conservative and aggressive decision characteristics of CCPs into GMCR and developed corresponding stability concepts; Zhang et al. [7] incorporated trust relationships among individuals within CCPs into GMCR, thereby constructing a social trust network GMCR; Liu et al. [26] incorporated community division into GMCR to identify power structures within CCPs and developed a unified preference representation for CCPs considering power differences.

The above literature indicates that GMCR has been substantially developed in preference modeling, preference elicitation, and the analysis of CCPs. In particular, option prioritization offers a structured way to elicit CCPs’ preferences through statement importance and state support. However, when GMCR is applied to complex conflict environments involving CCPs, preference elicitation becomes more demanding. On the one hand, existing option prioritization methods mainly use ordinal rankings to represent statement importance and binary evaluations to represent state support, which makes it difficult to capture nuanced differences in assessments. On the other hand, within a CCP, statement importance and state support may be assessed differently by different individuals. Although recent GMCR studies have examined CCPs from different perspectives, how to reconcile such heterogeneous individual assessments and construct collective preferences remains insufficiently explored.

**(2)**  **Consensus reaching process**

Decision making aims to provide analytical models and methodological tools for helping decision makers evaluate alternatives and select rational, effective, and acceptable courses of action under complex conditions [29,30,31]. Over time, decision making has developed into several major branches, including utility-based decision making [32], behavioral decision making [33], multiple criteria decision making [34], GDM [29] and so on.

Among them, GDM aims to find a collective solution to a decision problem in situations where a group of experts express their opinions regarding multiple alternatives [35]. Traditional research on GDM mainly focuses on preference aggregation rules [36], such as majority rule, plurality rule, and other social choice mechanisms. The pioneering achievements of Arrow, Sen, Simon, and others have greatly advanced the theoretical foundation and practical application of GDM research [30,37,38]. However, as decision environments become increasingly complex, many real-world problems require not only preference aggregation but also negotiation, assessment adjustment, and consensus formation [39]. Accordingly, the research paradigm of GDM has gradually shifted from preference aggregation to consensus negotiation, giving rise to CRP as an important research field.

Decision makers may express varying assessments of alternatives due to divergent value systems and knowledge backgrounds [40,41]. In this case, CRP is used to coordinate such expressed assessment information rather than to replace individuals’ fundamental preferences [39,42]. Specifically, individuals may revise their assessments under predefined consensus requirements, while minimum adjustment consensus models are employed to reduce information loss during the assessment adjustment process. Numerous minimum adjustment consensus models have been developed and widely applied in diverse decision-making settings. For example, Liu et al. [43] developed an opinion dynamics and minimum adjustment-driven consensus model for multi-criteria large-scale decision making. Li & Zhang [44] proposed a minimum adjustment consensus model for multicriteria group sorting problems based on the threshold-based value-driven sorting method. Li et al. [45] formulated a game-theoretic minimum adjustment consensus model that integrates a network game among decision makers with a Stackelberg game between the moderator and decision makers. Liu et al. [46] presented a minimum adjustment consensus model using mixed-integer programming that simultaneously achieves both cardinal and ordinal consensus in decision making.

Existing studies have developed numerous CRP models, particularly minimum adjustment consensus models, to manage preference divergences. However, the extension of CRP to the GMCR remains limited. In fact, when multiple individuals provide assessments within a CCP, assessment divergences are inevitable, which may compromise the validity of conflict analysis. To this end, it is necessary to incorporate CRP into the GMCR framework.

Based on the above literature review, the comparison of the proposed GMCR with the related GMCR studies is summarized in Table 1.

### 2.2. Basic Knowledge

#### 2.2.1. Graph Model for Conflict Resolution

**(1)**  **Structure of graph model**

In GMCR, conflict modeling requires the identification of several key elements, including conflicting parties, options, feasible states, state transitions, and preferences [7,15]. Let $P=\{P^{1},\ldots ,P^{k},\ldots ,P^{n}\}$ represent the set of n (n≥2) conflicting parties, and let N={1, 2, …, n}. For each k∈N, $P^{k}$ denotes the k-th conflicting party in P. Let $S=\{s_{1},\ldots ,s_{i},\ldots ,s_{h}\}$ denote the set of h (h≥2) feasible states, and let H={1, 2, …, h}. For each i∈H, $s_{i}$ represents the i-th feasible state, which corresponds to a feasible combination of options chosen by all conflicting parties. Let $A^{k}\subseteq S\times S$ (k∈N) denote the set of directed arcs controlled by $P^{k}$(k∈N). If $(s_{i},s_{j})\in A^{k}$, then $P^{k}$ can unilaterally move from state $s_{i}$ to $s_{j}$ in a single step. Each conflicting party $P^{k}$ has preferences over feasible states, which are characterized by $\left\{{≻}_{k},{∼}_{k}\right\}$. Specifically, $s_{i}{≻}_{k}s_{j}$ means that $P^{k}$ strictly prefers state $s_{i}$ to $s_{j}$, while $s_{i}{∼}_{k}s_{j}$ denotes that $P^{k}$ is indifferent between these two states. Accordingly, a graph model can be represented as $<P,\;S,\;{\left\{A^{k}\right\}}_{k\in N},{\left\{{≻}_{k},{∼}_{k}\right\}}_{k\in N}>$.

**(2)**  **Option prioritization**

Option prioritization is a useful preference elicitation technique in GMCR, through which conflicting parties provide an ordered set of option statements to rank their preferences over feasible states. In option prioritization, each statement takes a binary truth value, namely True (T) or False (F), to indicate whether it is satisfied in a feasible state [19]. Based on the statements and their truth values across feasible states, preferences over states can be determined.

Let $\Omega =\{\Omega_{1},\;\ldots ,\;\Omega_{j},\;\ldots ,\;\Omega_{k}\}$ denote the ordered set of *k* statements. The ordinal position of statements dictates their priority sequence. Let $\Omega_{j}(s_{i})\;(j=1,\;2,\;\ldots ,\;k)$ be the truth value of $\Omega_{j}$ at $s_{i}$. Specifically, for any two states $s_{i},\;s_{l}\in S$, $s_{i}\sim s_{l}$ if and only if (iff) $\Omega_{j}(s_{i})=\Omega_{j}(s_{l})$ for all j=1, 2, …, k. Moreover $s_{i}≻s_{l}$ iff either $\Omega_{1}(s_{i})=T$ and $\Omega_{1}(s_{l})=F$ or if there exists a *j* such that 0<j≤k and

$$\begin{array}{l} \Omega_{1}(s_{1})=\Omega_{1}(s_{2})\\ \Omega_{2}(s_{1})=\Omega_{2}(s_{2})\\ \;\vdots \\ \Omega_{j-1}(s_{1})=\Omega_{j-1}(s_{2})\\ \Omega_{j}(s_{1})=T\;\mathrm{and}\;\Omega_{j}(s_{2})=F. \end{array}$$

In addition, states can be ordered by a numerical score $\psi (s_{i})$ for conflicting parties. Let $\psi_{j}(s_{i})$ represent the increment value of state $s_{i}$ under $\Omega_{j}$. Define the increment value as $\psi_{j}(s_{i})=2^{k-j}$ when $\Omega_{j}(s_{i})=T$; otherwise, $\psi_{j}(s_{i})=0$. The score of $s_{i}$ is given by $\psi (s_{i})=\sum_{j=1}^{k}\psi_{j}(s_{i})$. Subsequently, states can be ordered according to their scores.

#### 2.2.2. Consensus Reaching Process

Let $P=\{P_{1},\ldots ,P_{l},\ldots ,P_{m}\}$ denote the set of *m* individuals. Let $\left\{V^{1},\ldots ,V^{l},\ldots ,V^{m}\right\}$ and $\left\{{\bar{V}}^{1},\ldots ,{\bar{V}}^{l},\ldots ,{\bar{V}}^{m}\right\}$ denote the original and adjusted assessments. The weight vector associated with P is denoted by $\lambda ={\left\{\lambda_{1},\;\lambda_{2},\;\ldots ,\;\lambda_{m}\right\}}^{T}$, where $\lambda_{l}\in [0,\;1]$ represents the weight assigned to $P_{l}$ and $\sum_{l=1}^{m}\lambda_{l}=1$. Let $F_{\lambda }(\cdot )$ be the aggregation function with respect to the weight vector λ, and let $CL({\bar{V}}^{1},{\bar{V}}^{2},\ldots ,{\bar{V}}^{m})$ denote the consensus level among the adjustment assessments. The predefined consensus threshold is denoted by α.

Let $d(V^{l},{\bar{V}}^{l})$ denote the deviation between the original and the adjusted assessments. To obtain optimal adjusted assessments, Dong et al. [47] proposed the following minimum adjustment consensus model:

(1) $$\begin{array}{l} \min\sum_{l=1}^{m}d(V^{l},{\bar{V}}^{l})\\ s.t.\left\{\begin{array}{l} {\bar{V}}^{c}=F_{\lambda }({\bar{V}}^{1},{\bar{V}}^{2},\ldots ,{\bar{V}}^{m})\\ CL({\bar{V}}^{1},{\bar{V}}^{2},\ldots ,{\bar{V}}^{m})\geq \alpha \end{array}\right. \end{array}.$$

In Model (1), the objective function minimizes the total deviation between $V^{l}$ and ${\bar{V}}^{l}$. The first constraint generates the adjusted collective assessment ${\bar{V}}^{c}$ by aggregating the adjusted individual assessments through $F_{\lambda }(\cdot )$. The second constraint requires the consensus level among the adjusted assessments to reach the predefined threshold.

#### 2.2.3. Pairwise Comparison

In the proposed framework, the main role of preference representation is to provide assessment information for CRP and subsequent GMCR stability analysis. The framework is not restricted to a single form of preference relation, as long as the adopted form can generate statement priority information for option prioritization. In this study, pairwise comparison is adopted to represent the relative importance among statements.

Pairwise comparison is a common way to express preference information by comparing objects two at a time [46,48]. This basic idea has been widely used in decision-making methods, such as the analytic hierarchy process, where pairwise judgments are usually expressed by a 1–9 scale. Similar to the pairwise comparison matrix constructed in analytic hierarchy process, the pairwise comparison relation used in this study is also presented in matrix form. The difference lies in the form of assessment information: analytic hierarchy process usually adopts a 1–9 scale, whereas this study uses numerical values within [0, 1] to represent the preference degree between two compared objects. Thus, pairwise comparison is used as a structured way to express statement priority information in GMCR option prioritization. Let $X=\{x_{1},x_{2},\ldots ,x_{g}\}$ denote the set of *g* alternatives. The concept of pairwise comparison over X, along with the corresponding consistency level, is introduced as follows.

**Definition** **1** ([49])**.*** The pairwise comparison over* X *is represented by a matrix* $F={\left(f_{ij}\right)}_{g\times g}$*, where *$f_{ij}\in [0,\;1]$* denotes the preference of alternative *$x_{i}$* over *$x_{j}$*. The elements of *$F={\left(f_{ij}\right)}_{g\times g}$* satisfy *$f_{ij}+f_{ji}=1$.

**Definition** **2** ([50])**.*** The consistency level of *$F={\left(f_{ij}\right)}_{g\times g}$* is measured below:*

(2) $$CI(F)=1-\frac{4}{g(g-1)(g-2)}\sum_{i,j,q=1;i<j<q}^{g}\left|\;f_{ij}+f_{jq}-f_{iq}-0.5\;\right|,$$

* where *CI(F)∈[0, 1].

The consistency level measures the internal coherence and transitivity of pairwise preference information [48]. Greater values of CI(F) indicate higher consistency, with CI(F)=1 implying complete consistency. Since complete consistency is difficult to achieve when many alternatives are involved, an acceptable consistency threshold is usually adopted [46]. Let β∈[0, 1] be the consistency threshold. The pairwise comparison matrix $F={\left(f_{ij}\right)}_{g\times g}$ is considered acceptable if CI(F)≥β, and unacceptable otherwise.

The priority vector derived from the pairwise comparison matrix $F={\left(f_{ij}\right)}_{g\times g}$ is denoted by $\omega ={\left(\omega_{1},\;\omega_{2},\;\ldots ,\;\omega_{g}\right)}^{T}$, where $\omega_{i}\in [0,\;1]$ represents the priority weight of alternative $x_{i}$ and $\sum_{i=1}^{g}\omega_{i}=1$. The value of $\omega_{i}$ is calculated as follows:

(3) $$\omega_{i}=\frac{2}{g^{2}-g}\sum_{j=1,j\neq i}^{g}f_{ij}.$$

In the GMCR framework, preferences over feasible states are constructed based on individuals’ assessments of statements. Thus, the representation of statement importance directly affects the resulting state preferences. In traditional option prioritization, the importance of statements is mainly reflected by their ordinal positions, and the satisfaction of each statement in a feasible state is usually represented by a binary truth value. This setting can identify the priority sequence of statements, but it does not provide detailed information about the relative preference degree between statements. Compared with ordinal rankings and binary evaluations used in traditional option prioritization, pairwise comparisons can capture not only the priority order of statements but also the relative degree of importance between statements through pairwise comparisons [46]. Therefore, to express statement importance in a more refined way, this study uses pairwise comparison over statements. Specifically, individuals compare statements two at a time and express the preference degree of one statement over another by a value within [0, 1]. These comparison values form a pairwise comparison matrix. When the matrix satisfies the acceptable consistency requirement, a priority vector can be derived to summarize the relative importance of statements. The derived priority vector is then used as statement priority information in the subsequent option prioritization process.

## 3. Complex Conflict Problem Formulation and Resolution Framework

This section formalizes the complex conflict problem and develops a resolution framework that integrates CRP into the GMCR framework.

### 3.1. Complex Conflict Problem Formulation

Within the complex conflict environment, there are multiple CCPs, denoted as $P=\{P^{1},\;\ldots ,P^{k},\;\ldots ,P^{n}\}$(n≥2). Each CCP $P^{k}$ is represented as $P^{k}=\{P_{1}^{k},\;\ldots ,P_{l}^{k},\;\ldots ,P_{m(k)}^{k}\}$, where $P_{l}^{k}$ represents an individual within $P^{k}$. Within the framework, each CCP $P^{k}$ controls a set of available options, denoted as $O^{k}=\{o_{1}^{k},\;o_{2}^{k},\;\ldots ,\;o_{h^{k}}^{k}\}$. A state represents a feasible combination of option selections made by all entities and can be represented as a vector of binary values. By excluding infeasible or inconsistent combinations, the set of h feasible states is obtained as $S=\{s_{1},\;s_{2},\;\ldots ,\;s_{h}\}$. Different strategic choices of options made by CCP $P^{k}$ can trigger transitions between conflict states, thereby forming a directed graph $D^{k}=(S,A^{k})$, where $A^{k}\subseteq S\times S$ denotes the set of directed arcs. If $(s_{i},s_{j})\in A^{k}$, then $P^{k}$ is able to move unilaterally from $s_{i}$ to $s_{j}$ in a single move. Let $\Omega^{k}=\{\Omega_{1}^{k},\;\Omega_{2}^{k},\;\ldots ,\;\Omega_{g(k)}^{k}\}$ denote the set of statements associated with CCP $P^{k}$, and let $G^{k}=\{1,\;2,\;\ldots ,\;g(k)\}$.

To capture such CCP divergences more precisely, flexible assessments are introduced to extend traditional option prioritization. Each individual $P_{l}^{k}$ provides two flexible assessments: the pairwise comparison matrix $F^{k,l}={\left(f_{ij}^{k,l}\right)}_{g(k)\times g(k)}$, which characterizes the relative importance of statements, and the flexible state assessment matrix $V^{k,l}={\left(v_{ij}^{k,l}\right)}_{h\times g(k)}$, which characterizes the support degree of each statement $\Omega_{j}^{k}$$\left(j\in G^{k}\right)$ at each state $s_{i}$(i∈H), where $v_{ij}^{k,l}\in [0,\;1]$. These two types of assessments provide the basis for deriving the CCP’s preferences over feasible states after the CRP.

### 3.2. Resolution Framework

Complex conflict problems typically exhibit a dual structural characteristic, involving inter-entity conflicts among CCPs and intra-entity assessment divergences within each CCP. To address these two aspects simultaneously, this study develops an integrated framework that incorporates CRP into GMCR. The proposed framework is organized as a stepwise process, in which intra-entity assessment divergences are resolved through CRP, and consensus-based preferences are incorporated into stability analysis. The detailed procedure is described as follows.

**Step 1. Conflict modeling.**

Conflict modeling is first conducted by identifying CCPs, individuals within each CCP, options, feasible states, state transitions, and statements. This step provides the basis for collecting individuals’ flexible assessments. Specifically, each individual provides two types of flexible assessments: a pairwise comparison matrix to express the relative importance of statements and a flexible state assessment matrix to describe the support degrees of statements across feasible states.

**Step 2. Conduct CRP for statement importance.**

For each CCP, the pairwise comparison matrices provided by individuals over statements are aggregated to generate a collective pairwise comparison matrix. The consensus level among individuals is then measured. If the consensus level is acceptable, the collective pairwise comparison matrix is retained as the consensual collective assessment of statement importance. Otherwise, a minimum adjustment consensus model with fairness concern is used to guide individuals in revising their pairwise comparison assessments. Through this process, a consensual collective pairwise comparison matrix over statements can be obtained for each CCP.

**Step 3. Conduct CRP for state support.**

Similarly, the flexible state assessment matrices provided by individuals are aggregated to generate a collective state assessment matrix, and the consensus level among individuals is measured. If the consensus level is unacceptable, a minimum adjustment consensus model with fairness concern is used to adjust individual state support assessments. This process continues until a consensual collective state assessment matrix is obtained for each CCP.

**Step 4. Derive CCPs’ preferences and conduct stability analysis.**

In Step 4, the information generated from the two CRPs is integrated to derive CCPs’ preferences over feasible states. Specifically, the consensual collective pairwise comparison matrix obtained in Step 2 is first transformed into statement priority information, which reflects the relative importance of statements. Meanwhile, the consensual collective state assessment matrix obtained in Step 3 describes the degree to which each statement is supported in each feasible state. By combining statement priority information with state support assessments, the preference values of feasible states are calculated for each CCP. These preference values are then used to rank feasible states and are incorporated into threshold-based stability analysis to identify equilibrium solutions.

**Figure 1:**
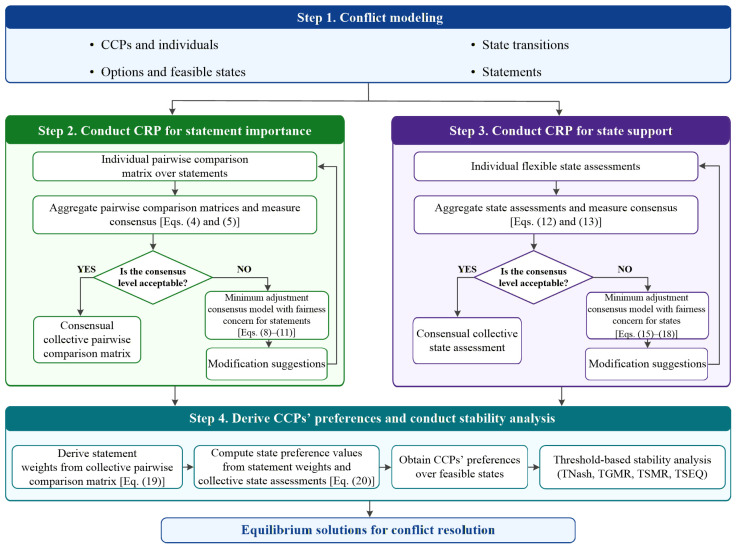
An integrated framework that incorporates consensus reaching process into graph model for conflict resolution.

It should be noted that the CRP in this framework does not aim to replace individuals’ fundamental preferences. Rather, it provides a structured process for coordinating the expressed assessment information provided by individuals within each CCP. The adjusted information mainly concerns individuals’ assessments of statement importance and state support under predefined consensus, consistency, and fairness requirements. Accordingly, the resulting consensus-based preferences should be understood as inputs for GMCR stability analysis. Overall, the proposed framework establishes a sequential link between intra-entity consensus reaching and inter-entity conflict analysis. The integrated framework is shown in Figure 1.

## 4. An Integrated Consensus-Based Framework for the Graph Model for Conflict Resolution

This section develops an integrated consensus-based framework for GMCR. In the proposed framework, two fairness-concerned consensus models based on flexible assessments are developed to resolve individuals’ assessment divergences. Following the paradigm of minimum adjustment consensus models in the existing CRP literature [44,46], the proposed models regard consensus and consistency as acceptability requirements for the adjusted assessments, while minimizing the total adjustment from the original assessments to reduce information loss. The obtained consensual collective assessments are used to derive the preferences of CCPs, which are then incorporated into threshold-based stability analysis to identify equilibrium solutions for conflict resolution.

### 4.1. Consensus Reaching Process for Resolving Individuals’ Assessment Divergences

#### 4.1.1. Consensus Reaching Process for Statement Importance

A fairness concern-driven CRP is employed to establish a consensual collective pairwise comparison matrix over statements of each CCP in this section.

**(1)**  **Consensus measure**

A fairness concern-driven CRP is employed to establish a consensual collective pairwise comparison matrix over statements of each CCP in this section. In this process, the pairwise comparison matrix represents individuals’ assessments of the relative importance of statements. The purpose of the CRP is to coordinate divergent pairwise comparison assessments among individuals within the same CCP, so that a consensual collective pairwise comparison matrix can be obtained and further used to derive statement priority information.

Within this framework, individual pairwise comparison matrices over the set of statements are provided for each CCP. The corresponding collective pairwise comparison matrix is then obtained by aggregating these individual assessments. Let $F^{k,l}={\left(f_{ij}^{k,l}\right)}_{g(k)\times g(k)}$ be as defined above. Let $F^{k,c}={\left(f_{ij}^{k,c}\right)}_{g(k)\times g(k)}$ denote the collective pairwise comparison matrix for $P^{k}$(k∈N), obtained from $\left\{F^{k,1},F^{k,2},\ldots ,F^{k,m(k)}\right\}$, where $f_{ij}^{k,c}$ is computed as:

(4) $$f_{ij}^{k,c}=\sum_{l=1}^{m(k)}\lambda_{l}^{k}\cdot f_{ij}^{k,l},$$

where $\lambda_{l}^{k}\in [0,\;1]$ indicates the weight of $P_{l}^{k}$, and $\sum_{l=1}^{m(k)}\lambda_{l}^{k}=1$.

The consensus level among $\left\{F^{k,1},F^{k,2},\ldots ,F^{k,m(k)}\right\}$ is computed as follows:

(5) $$CL(F^{k,1},\ldots ,F^{k,m(k)})=1-\frac{2}{m(k)\cdot g(k)\cdot [g(k)-1]}\sum_{l=1}^{m(k)}\sum_{i=1}^{g(k)-1}\sum_{j=i+1}^{g(k)}\left|\;f_{ij}^{k,l}-f_{ij}^{k,c}\;\right|,$$

where $CL(F^{k,1},\ldots ,F^{k,m(k)})\in [0,\;1]$. Larger value of $CL(F^{k,1},\ldots ,F^{k,m(k)})$ indicates a higher degree of consensus among individuals within $P^{k}$. As mentioned in Section 2.2, α is the predefined consensus level of $\left\{F^{k,1},F^{k,2},\ldots ,F^{k,m(k)}\right\}$ and a larger value of α imposes a stricter consensus requirement. The consensus level of all pairwise comparison matrices should meet or exceed the value of α. Then the consensual collective pairwise comparison matrix over statements of $P^{k}$ can be generated. Otherwise, the consensus level of $\left\{F^{k,1},F^{k,2},\ldots ,F^{k,m(k)}\right\}$ is deemed unacceptable, and individuals within CCPs are guided to adjust their initial assessments accordingly.

**(2)**  **Fairness concern**

During the CRP, individuals may compare their own assessment adjustments with those of others. Let ${\bar{F}}^{k,l}={\left({\bar{f}}_{ij}^{k,l}\right)}_{g(k)\times g(k)}$ be the adjusted assessment of individual $P_{l}^{k}$. The deviation of the adjusted assessment ${\bar{F}}^{k,l}$ from the original assessment $F^{k,l}$ is quantified by $d(F^{k,l},\;{\bar{F}}^{k,l})$. Then, individuals may be sensitive to unequal adjustment burdens during assessment modification, which is called fairness concern and has been investigated in GDM [51,52]. Specifically, when an individual’s adjustment is close to those of others, she/he perceives the process as fair. However, if an individual is required to make a substantially greater adjustment than others, a sense of unfairness may arise, and individuals may resist or even disrupt the consensus process to restore perceived psychological fairness. To quantify this effect, a fairness concern measure (FC) is proposed based on a fundamental behavioral logic: individuals assess fairness by comparing their own assessment adjustments with those of others:

(6) $$FC=\frac{2}{g(k)\cdot [g(k)-1]}\cdot [d(F^{k,l},\;{\bar{F}}^{k,l})-\frac{1}{m(k)-1}\sum_{t\in M^{k}/l}d(F^{k,t},\;{\bar{F}}^{k,t})]\;,\;l\in M^{k}$$

where $d(F^{k,l},\;{\bar{F}}^{k,l})=\sum_{i=1}^{g(k)-1}\sum_{j=i+1}^{g(k)}\left|\;f_{ij}^{k,l}-{\bar{f}}_{ij}^{k,l}\;\right|$ in this study.

Specifically, the deviation between an individual’s adjustment distance and the group average (excluding themselves) quantifies perceived unfairness. Usually, the lower the FC, the higher the level of fairness concern exhibited by the participants. Let ε∈[0, 1] be the upper bound of fairness-related deviations. It is not a predefined input parameter, but an endogenous decision variable to be optimized in the following model. Thus, during the process of reaching consensus, the fairness concern constraint should be met:

(7) $$\frac{2}{g(k)\cdot [g(k)-1]}\cdot (\sum_{i=1}^{g(k)-1}\sum_{j=i+1}^{g(k)}\left|\;f_{ij}^{k,l}-{\bar{f}}_{ij}^{k,l}\;\right|-\frac{1}{m(k)-1}\sum_{t\in M^{k}/l}\sum_{i=1}^{g(k)-1}\sum_{j=i+1}^{g(k)}\left|\;f_{ij}^{k,t}-{\bar{f}}_{ij}^{k,t}\;\right|)\leq \varepsilon \;,\;l\in M^{k}.$$

**(3)**  **Minimum adjustment consensus model with fairness concern for statements**

In CRP, the adjusted individual pairwise comparison matrix ${\bar{F}}^{k,l}={\left({\bar{f}}_{ij}^{k,l}\right)}_{g(k)\times g(k)}$ needs to be consistent, and its consistency level, $CI({\bar{F}}^{k,l})$, can be computed using Equation (2). When $CI({\bar{F}}^{k,l})=1$, ${\bar{F}}^{k,l}={\left({\bar{f}}_{ij}^{k,l}\right)}_{g(k)\times g(k)}$ is perfectly consistent. Let β denote the consistency threshold, where a larger value of β indicates a stricter requirement for consistency acceptability. The pairwise comparison matrix $F={\left(f_{ij}\right)}_{g\times g}$ is considered acceptable if CI(F)≥β; otherwise, it is unacceptable.

During the CRP, the fairness of the adjustment process is improved by minimizing ε. A smaller optimal value of ε indicates more balanced adjustment burdens among individuals. Therefore, the minimum adjustment consensus model with fairness concern for statements can be formulated as follows:

(8) $$\begin{array}{l} \min\;\varepsilon \\ s.t.\left\{\begin{array}{ll} {\bar{f}}_{ij}^{k,c}=\sum_{l=1}^{m(k)}\lambda_{l}^{k}\cdot {\bar{f}}_{ij}^{k,l},\;i,j\in G^{k},\;l\in M^{k} & \left(a\right)\\ \frac{2}{g(k)\cdot [g(k)-1]}\cdot (\sum_{i=1}^{g(k)-1}\sum_{j=i+1}^{g(k)}\left|\;f_{ij}^{k,l}-{\bar{f}}_{ij}^{k,l}\;\right|-\frac{1}{m(k)-1}\sum_{t\in M^{k}/l}\sum_{i=1}^{g(k)-1}\sum_{j=i+1}^{g(k)}\left|\;f_{ij}^{k,t}-{\bar{f}}_{ij}^{k,t}\;\right|)\leq \varepsilon \;,\;l\in M^{k}\; & \left(b\right)\\ 1-\frac{2}{m(k)\cdot g(k)\cdot [g(k)-1]}\sum_{l=1}^{m(k)}\sum_{i=1}^{g(k)-1}\sum_{j=i+1}^{g(k)}\left|\;{\bar{f}}_{ij}^{k,l}-{\bar{f}}_{ij}^{k,c}\;\right|\geq \alpha & \left(c\right)\\ 1-\frac{4}{g(k)[g(k)-1][g(k)-2]}\sum_{i,j,q=1;i<j<q}^{g(k)}\left|\;{\bar{f}}_{ij}^{k,l}+{\bar{f}}_{jq}^{k,l}-{\bar{f}}_{iq}^{k,l}-0.5\;\right|\geq \beta ,\;l\in M^{k} & \left(d\right)\\ \sum_{l=1}^{m(k)}\sum_{i=1}^{g(k)-1}\sum_{j=i+1}^{g(k)}\left|\;f_{ij}^{k,l}-{\bar{f}}_{ij}^{k,l}\;\right|=D & \left(e\right)\\ \;{\bar{f}}_{ij}^{k,l},\;{\bar{f}}_{ij}^{k,c},\;\varepsilon \in [0,\;1],\;i,\;j\in G^{k},\;l\in M^{k} & \left(f\right) \end{array}\right. \end{array},$$

where ${\bar{F}}^{k,l}={\left({\bar{f}}_{ij}^{k,l}\right)}_{g(k)\times g(k)}$, ${\bar{F}}^{k,c}={\left({\bar{f}}_{ij}^{k,c}\right)}_{g(k)\times g(k)}$, and ε are decision variables, and D represents the total modification adjustment of all individuals.

Since information loss may occur during the CRP, a minimum adjustment consensus model is proposed to reduce such loss.

(9) $$\begin{array}{l} \min\sum_{l=1}^{m(k)}\sum_{i=1}^{g(k)-1}\sum_{j=i+1}^{g(k)}\left|\;f_{ij}^{k,l}-{\bar{f}}_{ij}^{k,l}\;\right|\\ s.t.\left\{\begin{array}{ll} {\bar{f}}_{ij}^{k,c}=\sum_{l=1}^{m(k)}\lambda_{l}^{k}\cdot {\bar{f}}_{ij}^{k,l},\;i,j\in G^{k},\;l\in M^{k} & \left(a\right)\\ 1-\frac{2}{m(k)\cdot g(k)\cdot [g(k)-1]}\sum_{l=1}^{m(k)}\sum_{i=1}^{g(k)-1}\sum_{j=i+1}^{g(k)}\left|\;{\bar{f}}_{ij}^{k,l}-{\bar{f}}_{ij}^{k,c}\;\right|\geq \alpha & \left(b\right)\\ 1-\frac{4}{g(k)[g(k)-1][g(k)-2]}\sum_{i,j,q=1;i<j<q}^{g(k)}\left|\;{\bar{f}}_{ij}^{k,l}+{\bar{f}}_{jq}^{k,l}-{\bar{f}}_{iq}^{k,l}-0.5\;\right|\geq \beta ,\;l\in M^{k}\; & \left(c\right)\\ \;{\bar{f}}_{ij}^{k,l},{\bar{f}}_{ij}^{k,c}\in [0,\;1],\;i,j\in G^{k},\;l\in M^{k} & \left(d\right) \end{array}\right. \end{array}.$$

For ease of reference, models (8) and (9) are denoted as $M_{1}$ and $M_{2}$, respectively. To reduce computational complexity, the original model $M_{2}$ is transformed into an equivalent linear programming model $M_{2}\prime$ by introducing auxiliary variables and linearization techniques. This transformation reformulates nonlinear terms into linear constraints, enabling the model to be efficiently solved using the CPLEX solver.

This paper proposes a minimum adjustment-based CRP to obtain the importance of statements. For future research, it would be valuable to explore how classical axiomatic foundations of weight determination, such as those derived from indifference conditions in utility theory [53], can be integrated into statement evaluation within the graph model.

**Proposition** **1.** *By introducing new variables *$a_{ij}^{k,l}$*, *$b_{ij}^{k,l}$*, and *$c_{ijq}^{k,l}$*, the original model* $M_{2}$* can be transformed into an equivalent linear programming model *$M_{2}\prime$.

(10) $$\begin{array}{l} \min\sum_{l=1}^{m(k)}\sum_{i=1}^{g(k)-1}\sum_{j=i+1}^{g(k)}a_{ij}^{k,l}\\ s.t.\left\{\begin{array}{ll} a_{ij}^{k,l}\geq f_{ij}^{k,l}-{\bar{f}}_{ij}^{k,l},\;i,j\in G^{k},\;l\in M^{k} & \left(a\right)\\ a_{ij}^{k,l}\geq {\bar{f}}_{ij}^{k,l}-f_{ij}^{k,l},\;i,j\in G^{k},\;l\in M^{k} & \left(b\right)\\ {\bar{f}}_{ij}^{k,c}=\sum_{l=1}^{m(k)}\lambda_{l}^{k}\cdot {\bar{f}}_{ij}^{k,l},\;i,j\in G^{k},\;l\in M^{k} & \left(c\right)\\ 1-\frac{2}{m(k)\cdot g(k)\cdot [g(k)-1]}\sum_{l=1}^{m(k)}\sum_{i=1}^{g(k)-1}\sum_{j=i+1}^{g(k)}b_{ij}^{k,l}\geq \alpha & \left(d\right)\\ b_{ij}^{k,l}\geq {\bar{f}}_{ij}^{k,l}-{\bar{f}}_{ij}^{k,c},\;i,j\in G^{k},\;l\in M^{k} & (e).\\ b_{ij}^{k,l}\geq {\bar{f}}_{ij}^{k,c}-{\bar{f}}_{ij}^{k,l},\;i,j\in G^{k},\;l\in M^{k} & \left(f\right)\\ 1-\frac{4}{g(k)[g(k)-1][g(k)-2]}\sum_{i,j,q=1;i<j<q}^{g(k)}c_{ijq}^{k,l}\geq \beta \;,\;l\in M^{k} & \left(g\right)\\ c_{ijq}^{k,l}\geq {\bar{f}}_{ij}^{k,l}+{\bar{f}}_{jq}^{k,l}-{\bar{f}}_{iq}^{k,l}-0.5,\;i,j,q\in G^{k},\;l\in M^{k} & \left(h\right)\\ c_{ijq}^{k,l}\geq -{\bar{f}}_{ij}^{k,l}-{\bar{f}}_{jq}^{k,l}+{\bar{f}}_{iq}^{k,l}+0.5,\;i,j,q\in G^{k},\;l\in M^{k} & \left(i\right)\\ {\bar{f}}_{ij}^{k,l},{\bar{f}}_{ij}^{k,c},a_{ij}^{k,l},\;b_{ij}^{k,l}\in [0,\;1],\;c_{ijq}^{k,l}\in [0,\;1.5],\;i,j,q\in G^{k},\;l\in M^{k}\; & \left(j\right) \end{array}\right. \end{array}$$

**Proof.**  In $M_{2}^{\prime }$, constraints (*a*) and (*b*) imply that $a_{ij}^{k,l}\geq |\;f_{ij}^{k,l}-{\bar{f}}_{ij}^{k,l}\;|$. Considering the objective function aims to minimize adjustment, the objective function is taken to be optimal when $a_{ij}^{k,l}=|\;f_{ij}^{k,l}-{\bar{f}}_{ij}^{k,l}\;|$. Thus, the objective function of $M_{2}$ can be equivalently converted into the objective function of $M_{2}\prime$, constraints (*a*) and (*b*). Constraints (*e*) and (*f*) in $M_{2}\prime$ imply that $b_{ij}^{k,l}\geq |\;{\bar{f}}_{ij}^{k,l}-{\bar{f}}_{ij}^{k,c}\;|$. Further, considering constraint (*d*), we have $1-\frac{2}{m(k)\cdot g(k)\cdot [g(k)-1]}\sum_{k=1}^{m(k)}\sum_{i=1}^{g(k)-1}\sum_{j=i+1}^{g(k)}\left|\;{\bar{f}}_{ij}^{k,l}-{\bar{f}}_{ij}^{k,c}\;\right|\geq 1-\frac{2}{m(k)\cdot g(k)\cdot [g(k)-1]}\sum_{k=1}^{m(k)}\sum_{i=1}^{g(k)-1}\sum_{j=i+1}^{g(k)}b_{ij}^{k}\geq \alpha$. Thus, constraints (*d*), (*e*) and (*f*) in $M_{2}\prime$ are equivalent to constraint (*b*) in $M_{2}$. Constraints (*h*) and (*i*) in $M_{2}\prime$ imply that $c_{ijq}^{k,l}\geq |\;{\bar{f}}_{ij}^{k,l}+{\bar{f}}_{jq}^{k,l}-{\bar{f}}_{iq}^{k,l}-0.5\;|$. Thus, $1-\frac{4}{g(k)[g(k)-1][g(k)-2]}\sum_{i,j,q=1;i<j<q}^{g(k)}\left|\;{\bar{f}}_{ij}^{k,l}+{\bar{f}}_{jq}^{k,l}-{\bar{f}}_{iq}^{k,l}-0.5\;\right|\geq 1-\frac{4}{g(k)[g(k)-1][g(k)-2]}\sum_{i,j,q=1;i<j<q}^{g(k)}c_{ijq}^{k,l}\geq \beta$ holds. Thus, constraints (*g*), (*h*) and (*i*) in $M_{2}\prime$ are equivalent to constraint (*c*) in $M_{2}$. Accordingly, $M_{2}$ can be converted to linear programming model $M_{2}\prime$ equivalently. □

By solving model (10), the optimal solution can be obtained, which represents the total minimum adjustment amount and is denoted as $D^{*}$. Accordingly, model $M_{1}$ can be reformulated as an equivalent linear programming formulation, denoted as $M_{1}\prime$.

**Proposition** **2.** *When the constant *D* in model *$M_{1}$* is set to *$D^{*}$, model $M_{1}$* can be reformulated as the following linear programming model *$M_{1}\prime$.

(11) $$\begin{array}{l} \min\;\varepsilon \\ s.t.\left\{\begin{array}{ll} {\bar{f}}_{ij}^{k,c}=\sum_{l=1}^{m(k)}\lambda_{l}^{k}\cdot {\bar{f}}_{ij}^{k,l},\;i,j\in G^{k},\;l\in M^{k} & \left(a\right)\\ \frac{2}{g(k)\cdot [g(k)-1]}\cdot (\sum_{i=1}^{g(k)-1}\sum_{j=i+1}^{g(k)}a_{ij}^{k,l}-\frac{1}{m(k)-1}\sum_{t\in M^{k}/l}\sum_{i=1}^{g(k)-1}\sum_{j=i+1}^{g(k)}a_{ij}^{k,t})\leq \varepsilon \;,\;l\in M^{k}\; & \left(b\right)\\ a_{ij}^{k,l}\geq f_{ij}^{k,l}-{\bar{f}}_{ij}^{k,l},\;i,j\in G^{k},\;l\in M^{k} & \left(c\right)\\ a_{ij}^{k,l}\geq -f_{ij}^{k,l}+{\bar{f}}_{ij}^{k,l},\;i,j\in G^{k},\;l\in M^{k} & \left(d\right)\\ 1-\frac{2}{m(k)\cdot g(k)\cdot [g(k)-1]}\sum_{l=1}^{m(k)}\sum_{i=1}^{g(k)-1}\sum_{j=i+1}^{g(k)}b_{ij}^{k,l}\geq \alpha & \left(e\right)\\ b_{ij}^{k,l}\geq {\bar{f}}_{ij}^{k,l}-{\bar{f}}_{ij}^{k,c},\;i,j\in G^{k},\;l\in M^{k} & \left(f\right)\\ b_{ij}^{k,l}\geq {\bar{f}}_{ij}^{k,c}-{\bar{f}}_{ij}^{k,l},\;i,j\in G^{k},\;l\in M^{k} & \left(g\right)\\ 1-\frac{4}{g(k)[g(k)-1][g(k)-2]}\sum_{i,j,q=1;i<j<q}^{g(k)}c_{ijq}^{k,l}\geq \beta ,\;l\in M^{k} & \left(h\right)\\ c_{ijq}^{k,l}\geq {\bar{f}}_{ij}^{k,l}+{\bar{f}}_{jq}^{k,l}-{\bar{f}}_{iq}^{k,l}-0.5,\;i,j,q\in G^{k},\;l\in M^{k} & \left(i\right)\\ c_{ijq}^{k,l}\geq -{\bar{f}}_{ij}^{k,l}-{\bar{f}}_{jq}^{k,l}+{\bar{f}}_{iq}^{k,l}+0.5,\;i,j,q\in G^{k},\;l\in M^{k} & \left(j\right)\\ \sum_{l=1}^{m(k)}\sum_{i=1}^{g(k)-1}\sum_{j=i+1}^{g(k)}a_{ij}^{k,l}=D & \left(k\right)\\ {\bar{f}}_{ij}^{k,l},{\bar{f}}_{ij}^{k,c},a_{ij}^{k,l},\;b_{ij}^{k,l},\;\varepsilon \in [0,\;1],\;c_{ijq}^{k,l}\in [0,\;1.5],\;i,j,q\in G^{k},\;l\in M^{k} & (l). \end{array}\right. \end{array}$$

**Proof.**  In $M_{1}\prime$, constraints (*c*) and (*d*) imply that $a_{ij}^{k,l}\geq |\;f_{ij}^{k,l}-{\bar{f}}_{ij}^{k,l}\;|$. Meanwhile, in $M_{1}\prime$, $D=D^{*}$, where $D^{*}$ represents the optimal solution of $M_{2}$, $D^{*}=\min\sum_{l=1}^{m(k)}\sum_{i=1}^{g(k)-1}\sum_{j=i+1}^{g(k)}\left|\;f_{ij}^{k,l}-{\bar{f}}_{ij}^{k,l}\;\right|$. Assume that there exists $a_{ij}^{k,l}>|\;f_{ij}^{k,l}-{\bar{f}}_{ij}^{k,l}\;|$. To maintain $D=D^{*}$, it is necessary that there exists another $a_{ij}^{k,l}<|\;f_{ij}^{k,l}-{\bar{f}}_{ij}^{k,l}\;|$. However, $a_{ij}^{k,l}<|\;f_{ij}^{k,l}-{\bar{f}}_{ij}^{k,l}\;|$ violates constraints (*c*) and (*d*) in $M_{1}\prime$. Therefore, we have $a_{ij}^{k,l}=|\;f_{ij}^{k,l}-{\bar{f}}_{ij}^{k,l}\;|$. It follows that constraints (*b*), (*c*) and (*d*) in $M_{1}\prime$ are equivalent to constraint (*b*) in $M_{1}$, and constraint (*k*) in $M_{1}\prime$ is equivalent to constraint (*e*) in $M_{1}$. Next, constraints (*f*), (*g*) of $M_{1}\prime$ imply that $b_{ij}^{k,l}\geq |\;{\bar{f}}_{ij}^{k,l}-{\bar{f}}_{ij}^{k,c}\;|$. Based on constraint (*e*), we have $1-\frac{2}{m(k)\cdot g(k)\cdot [g(k)-1]}\sum_{k=1}^{m(k)}\sum_{i=1}^{g(k)-1}\sum_{j=i+1}^{g(k)}|\;{\bar{f}}_{ij}^{k,l}-{\bar{f}}_{ij}^{k,c}\;|\geq 1-\frac{2}{m(k)\cdot g(k)\cdot [g(k)-1]}\sum_{k=1}^{m(k)}\sum_{i=1}^{g(k)-1}\sum_{j=i+1}^{g(k)}b_{ij}^{k}\geq \alpha$ holds. Thus, constraints (*e*), (*f*) and (*g*) in $M_{1}\prime$ are equivalent to constraint (*c*) in $M_{1}$. According to constraints (*i*) and (*j*) of $M_{1}\prime$, it is $c_{ij}^{k,l}\geq |\;{\bar{f}}_{ij}^{k,l}+{\bar{f}}_{jq}^{k,l}-{\bar{f}}_{iq}^{k,l}-0.5\;|$. Hence, $1-\frac{4}{g(k)[g(k)-1][g(k)-2]}\sum_{i,j,q=1;i<j<q}^{g(k)}\left|\;{\bar{f}}_{ij}^{k,l}+{\bar{f}}_{jq}^{k,l}-{\bar{f}}_{iq}^{k,l}-0.5\;\right|\geq 1-\frac{4}{g(k)[g(k)-1][g(k)-2]}\times \sum_{i,j,q=1;i<j<q}^{g(k)}c_{ijq}^{k,l}\geq \beta$ holds. Thus, constraints (*h*), (*i*) and (*j*) in $M_{1}\prime$ are equivalent to constraint (*d*) in $M_{1}$. Therefore, $M_{1}$ can be converted to linear programming model $M_{1}\prime$ equivalently. □

It is worth noting that when setting ${\bar{f}}_{ij}^{k,l}=0.5$ ($i,j\in G^{k},\;l\in M^{k}$), all constraints of model (9) are satisfied, indicating that the feasible region of model (9) is nonempty. Furthermore, both the objective function and constraints of model (9) are bounded, ensuring the existence of multiple feasible solutions. Moreover, model (8) is used to identify the feasible solution with the highest degree of fairness concern among the optimal solutions of model (9). Therefore, model (8) also has at least one feasible solution.

#### 4.1.2. Consensus Reaching Process for State Support

A fairness concern-driven CRP is employed to establish a consensual collective flexible state assessment matrix of each CCP in this section.

**(1)**  **Consensus measure**

Within this framework, the flexible state assessment matrices provided by individual CCP members are aggregated to construct a collective assessment. Let $V^{k,l}={\left(v_{ij}^{k,l}\right)}_{h\times g(k)}$ be as defined above. Let $V^{k,c}={\left(v_{ij}^{k,c}\right)}_{h\times g(k)}$ be the collective flexible state assessment matrix of $P^{k}$, obtained from $\left\{V^{k,1},V^{k,2},\ldots ,V^{k,m(k)}\right\}$, where

(12) $$v_{ij}^{c,k}=\sum_{l=1}^{m(k)}\lambda_{l}^{k}\cdot v_{ij}^{k,l}.$$

The consensus level among $\left\{V^{k,1},V^{k,2},\ldots ,V^{k,m(k)}\right\}$ is computed as follows:

(13) $$CL(V^{k,1},\;\ldots ,\;V^{k,m(k)})=1-\frac{1}{m(k)\cdot h\cdot g(k)}\sum_{l=1}^{m(k)}\sum_{i=1}^{h}\sum_{j=1}^{g(k)}\left|\;v_{ij}^{k,l}-v_{ij}^{k,c}\;\right|.$$

The consensus level of $\left\{V^{k,1},V^{k,2},\ldots ,V^{k,m(k)}\right\}$ is required to meet or exceed the predefined consensus threshold α. In this case, the consensual collective flexible state assessment matrix of $P^{k}$ can be generated; otherwise, individuals within the CCPs are guided to adjust their initial assessments accordingly.

**(2)**  **Fairness concern**

Let ${\bar{V}}^{k,l}={\left({\bar{v}}_{ij}^{k,l}\right)}_{h\times g(k)}$ the adjusted flexible state assessment matrix of individual $P_{l}^{k}$. During the assessment modification process, individuals naturally tend to compare their own assessment adjustments with those of others. To account for this, all assessment modifications must satisfy the following fairness concern constraint:

(14) $$\frac{1}{h\cdot g(k)}\cdot (\sum_{i=1}^{h}\sum_{j=1}^{g(k)}\left|\;v_{ij}^{k,l}-{\bar{v}}_{ij}^{k,l}\;\right|-\frac{1}{m(k)-1}\sum_{t\in M^{k}/l}\sum_{i=1}^{h}\sum_{j=1}^{g(k)}\left|\;v_{ij}^{k,t}-{\bar{v}}_{ij}^{k,t}\;\right|)\leq \varepsilon ,l\in M^{k}.$$

**(3)**  **Minimum adjustment consensus model with fairness concern for states**

Let ${\bar{V}}^{k,c}={\left({\bar{v}}_{ij}^{k,c}\right)}_{h\times g(k)}$ be the adjusted collective flexible state assessment matrix by aggregating the adjusted state assessment matrices. It is evident that the consensus level of the state assessment matrices $\left\{{\bar{V}}^{k,1},\;{\bar{V}}^{k,2},\;\ldots ,\;{\bar{V}}^{k,m(k)}\right\}$ is expected to be acceptable, where $CL({\bar{V}}^{k,1},\ldots ,{\bar{V}}^{k,m(k)})\geq \alpha$. When addressing consensus, the overall fairness level should be maximized. A lower value of ε signifies a higher degree of fairness concern among participants. Accordingly, the minimum adjustment consensus model with fairness concern for states can be formulated as follows:

(15) $$\begin{array}{l} \min\;\varepsilon \\ s.t.\left\{\begin{array}{ll} {\bar{v}}_{ij}^{k,c}=\sum_{l=1}^{m(k)}\lambda_{l}^{k}\cdot {\bar{v}}_{ij}^{k,l},\;l\in M^{k},\;i\in H,\;j\in G^{k} & \left(a\right)\\ \frac{1}{h\cdot g(k)}\cdot (\sum_{i=1}^{h}\sum_{j=1}^{g(k)}\left|\;v_{ij}^{k,l}-{\bar{v}}_{ij}^{k,l}\;\right|-\frac{1}{m(k)-1}\sum_{t\in M^{k}/l}\sum_{i=1}^{h}\sum_{j=1}^{g(k)}\left|\;v_{ij}^{k,t}-{\bar{v}}_{ij}^{k,t}\;\right|)\leq \varepsilon ,l\in M^{k}\; & \left(b\right)\\ 1-\frac{1}{m(k)\cdot h\cdot g(k)}\sum_{l=1}^{m(k)}\sum_{i=1}^{h}\sum_{j=1}^{g(k)}\left|\;{\bar{v}}_{ij}^{k,l}-{\bar{v}}_{ij}^{k,c}\;\right|\geq \alpha ,\;l\in M^{k} & \left(c\right)\\ \sum_{l=1}^{m(k)}\sum_{i=1}^{h}\sum_{j=1}^{g(k)}\left|\;v_{ij}^{k,l}-{\bar{v}}_{ij}^{k,l}\;\right|=D & \left(d\right)\\ {\bar{v}}_{ij}^{k,l},\;{\bar{v}}_{ij}^{k,c},\;\varepsilon \in [0,\;1],\;l\in M^{k},\;i\in H,\;j\in G^{k} & \left(e\right) \end{array}\right. \end{array},$$

where $V^{k,l}={\left(v_{ij}^{k,l}\right)}_{h\times g(k)}$, ${\bar{V}}^{k,c}={\left({\bar{v}}_{ij}^{k,c}\right)}_{h\times g(k)}$, and ε are decision variables, D represents the total modification adjustment of all individuals.

The following minimum adjustment consensus model is presented in order to address information loss during the CRP:

(16) $$\begin{array}{l} \min\sum_{l=1}^{m(k)}\sum_{i=1}^{h}\sum_{j=1}^{g(k)}\left|\;v_{ij}^{k,l}-{\bar{v}}_{ij}^{k,l}\;\right|\\ s.t.\left\{\begin{array}{ll} {\bar{v}}_{ij}^{k,c}=\sum_{l=1}^{m(k)}\lambda_{l}^{k}\cdot {\bar{v}}_{ij}^{k,l},\;i\in H,\;j\in G^{k},\;l\in M^{k} & \left(a\right)\\ 1-\frac{1}{m(k)\cdot h\cdot g(k)}\sum_{l=1}^{m(k)}\sum_{i=1}^{h}\sum_{j=1}^{g(k)}\left|\;{\bar{v}}_{ij}^{k,l}-{\bar{v}}_{ij}^{k,c}\;\right|\geq \alpha ,\;l\in M^{k}\; & \left(b\right)\\ {\bar{v}}_{ij}^{k,l},{\bar{v}}_{ij}^{k,c},\in [0,\;1],\;i\in H,\;j\in G^{k},\;l\in M^{k} & \left(c\right) \end{array}\right. \end{array}$$

For convenience, model (15) and model (16) are denoted as $M_{3}$ and $M_{4}$, respectively. The following propositions present the equivalent linear programming formulations of $M_{3}$ and $M_{4}$ through the introduction of auxiliary variables.

**Proposition** **3.** *By introducing new variables *$a_{ij}^{k,l}$* and *$b_{ij}^{k,l}$*, model *$M_{4}$* is equivalently converted into the linear programming model *$M_{4}\prime$.

(17) $$\begin{array}{l} \min\sum_{l=1}^{m(k)}\sum_{i=1}^{h}\sum_{j=1}^{g(k)}a_{ij}^{k,l}\\ s.t.\left\{\begin{array}{ll} a_{ij}^{k,l}\geq v_{ij}^{k,l}-{\bar{v}}_{ij}^{k,l},\;i\in H,\;j\in G^{k},\;l\in M^{k} & \left(a\right)\\ a_{ij}^{k,l}\geq {\bar{v}}_{ij}^{k,l}-v_{ij}^{k,l},\;i\in H,\;j\in G^{k},\;l\in M^{k} & \left(b\right)\\ {\bar{v}}_{ij}^{k,c}=\sum_{l=1}^{m(k)}\lambda_{l}^{k}\cdot {\bar{v}}_{ij}^{k,l},\;i\in H,\;j\in G^{k},\;l\in M^{k} & \left(c\right)\\ 1-\frac{1}{m(k)\cdot h\cdot g(k)}\sum_{l=1}^{m(k)}\sum_{i=1}^{h}\sum_{j=1}^{g(k)}b_{ij}^{k,l}\geq \alpha ,\;l\in M^{k} & \left(d\right)\\ b_{ij}^{k,l}\geq {\bar{v}}_{ij}^{k,l}-{\bar{v}}_{ij}^{k,c},\;i\in H,\;j\in G^{k},\;l\in M^{k} & \left(e\right)\\ b_{ij}^{k,l}\geq {\bar{v}}_{ij}^{k,c}-{\bar{v}}_{ij}^{k,l},\;i\in H,\;j\in G^{k},\;l\in M^{k} & \left(f\right)\\ {\bar{v}}_{ij}^{k,l},{\bar{v}}_{ij}^{k,c},a_{ij}^{k,l},\;b_{ij}^{k,l}\in [0,\;1],\;i\in H,\;j\in G^{k},\;l\in M^{k} & \left(g\right) \end{array}\right. \end{array}.$$

We omit the proof of Proposition 3 to conserve space, as it follows a similar structure to that of Proposition 1.

The optimal value derived from solving model $M_{4}\prime$ is denoted as $D^{*}$, which quantifies the total minimum adjustment amount.

**Proposition** **4.** *When the constant *D* in model *$M_{3}$* is set to *$D^{*}$, model $M_{3}$* an be converted into the following linear programming model *$M_{3}\prime$.

(18) $$\begin{array}{l} \min\;\varepsilon \\ s.t.\left\{\begin{array}{ll} {\bar{v}}_{ij}^{k,c}=\sum_{l=1}^{m(k)}\lambda_{l}^{k}\cdot {\bar{v}}_{ij}^{k,l},\;i\in H,\;j\in G^{k},\;l\in M^{k} & \left(a\right)\\ \frac{1}{h\cdot g(k)}\cdot (\sum_{i=1}^{h}\sum_{j=1}^{g(k)}a_{ij}^{k,l}-\frac{1}{m(k)-1}\sum_{t\in M^{k}/l}\sum_{i=1}^{h}\sum_{j=1}^{g(k)}a_{ij}^{k,t})\leq \varepsilon ,\;l\in M^{k} & \left(b\right)\\ a_{ij}^{k,l}\geq v_{ij}^{k,l}-{\bar{v}}_{ij}^{k,l},\;i\in H,\;j\in G^{k},\;l\in M^{k} & \left(c\right)\\ a_{ij}^{k,l}\geq {\bar{v}}_{ij}^{k,l}-v_{ij}^{k,l},\;i\in H,\;j\in G^{k},\;l\in M^{k} & \left(d\right)\\ 1-\frac{1}{m(k)\cdot h\cdot g(k)}\sum_{l=1}^{m(k)}\sum_{i=1}^{h}\sum_{j=1}^{g(k)}b_{ij}^{k,l}\geq \alpha ,\;l\in M^{k} & \left(e\right)\\ b_{ij}^{k,l}\geq {\bar{v}}_{ij}^{k,l}-{\bar{v}}_{ij}^{k,c},\;i\in H,\;j\in G^{k},\;l\in M^{k} & \left(f\right)\\ b_{ij}^{k,l}\geq {\bar{v}}_{ij}^{k,c}-{\bar{v}}_{ij}^{k,l},\;i\in H,\;j\in G^{k},\;l\in M^{k} & \left(g\right)\\ \sum_{l=1}^{m(k)}\sum_{i=1}^{h}\sum_{j=1}^{g(k)}a_{ij}^{k,l}=D & \left(h\right)\\ {\bar{v}}_{ij}^{k,l},\;{\bar{v}}_{ij}^{k,c},\;a_{ij}^{k,l},\;b_{ij}^{k,l},\;\varepsilon \in [0,\;1],\;i\in H,\;j\in G^{k},\;l\in M^{k} & \left(i\right) \end{array}\right. \end{array}.$$

We omit the proof of Proposition 4 to conserve space, as it follows a similar structure to that of Proposition 2.

Similarly, the solution properties of models (15) and (16) can be analyzed in the same manner as those of models (8) and (9), respectively. Therefore, they are not elaborated further here.

### 4.2. Stability Analysis Based on Preference Threshold for Conflict Resolution

Solving models (8) and (15), the consensual collective pairwise comparison matrix for statement importance and the consensual collective flexible state assessment matrix for state support are obtained. These assessments are then used to derive state preference scores, which serve as the basis for the subsequent GMCR stability analysis.

Let $\omega^{k}={\left(\omega_{1}^{k},\omega_{2}^{k},\ldots ,\omega_{g(k)}^{k}\right)}^{T}$ be the priority vector of statements for $P^{k}$, where $\omega_{i}^{k}$ denotes the weight of statement $\Omega_{i}^{k}$, computed as:

(19) $$\omega_{i}^{k}=\frac{2}{g{\left(k\right)}^{2}-g(k)}\sum_{j=1,j\neq i}^{g(k)}{\bar{f}}_{ij}^{k,c}.$$

The priority vector represents the relative importance of statements, while the flexible state assessment matrix captures the degree to which each statement is supported in each feasible state. The preference score of a feasible state therefore depends not only on the importance of statements, but also on the degree to which these statements are supported in that state. A state receives a higher preference score when it is more strongly supported by more important statements. Based on the priority vector $\omega^{k}$ and ${\bar{V}}^{k,c}={\left({\bar{v}}_{ij}^{k,c}\right)}_{h\times g(k)}$, the preference vector $PV^{k}={\left(pv_{1}^{k},\;pv_{2}^{k},\;\ldots ,\;pv_{h}^{k}\right)}^{T}$ can be obtained. Here, $pv_{i}^{k}$ reflects the preference of state $s_{i}$ provided by $P^{k}$, which can be computed as follows:

(20) $$pv_{i}^{k}=\sum_{j=1}^{g(k)}\omega_{j}^{k}\cdot {\bar{v}}_{ij}^{k,c},i=1,2,\ldots ,h;j=1,2,\ldots ,g(k).$$

This score provides a deterministic basis for ranking feasible states and conducting subsequent GMCR stability analysis. In the GMCR framework, stability analysis evaluates the stability of a state based on different stability concepts. This study employs the preference threshold-based stability concepts introduced by Chen et al. [10]. Let $R^{k}(s_{i})$ denote the reachable set of unilateral moves, comprising all states that $P^{k}$ can reach from $s_{i}\in S$ in one step. Let $R^{k,+}(s_{i})$ denote the reachable set of unilateral improvements, which includes all states that $P^{k}$ can move to from $s_{i}\in S$ in one step, and that $P^{k}$ strictly prefers to $s_{i}$. Specifically, a state $s_{j}\in R^{k}(s_{i})$ is included in $R^{k,+}(s_{i})$ if it satisfies the condition $pv_{j}^{k}\geq pv_{i}^{k}+\gamma^{k}$, where $\gamma^{k}\in [0,\;1]$ is a preference threshold for significant utility differences.

Behaviorally, $\gamma^{k}$ represents the minimum utility increment required to trigger a unilateral improvement, reflecting CCPs’ bounded rationality and switching costs. However, determining an appropriate value for $\gamma^{k}$ is a practical challenge. Effectively calibrating this threshold depends heavily on the specific characteristics of the given conflict scenario, which is beyond the scope of this study. Future research could explore empirical calibration procedures or data-driven techniques to systematically determine these preference parameters. The preference threshold-based stability concepts [10], including threshold-based Nash (TNash), threshold-based GMR (TGMR), threshold-based SMR (TSMR), and threshold-based SEQ (TSEQ), are formally defined as follows.

**Definition 3** (TNash)**.** *For *$P^{k}\;(k\in N)$*, the state *$s_{i}\in S$* is regarded as TNash stability iff *$R^{k,+}(s_{i})=\emptyset$.

**Definition 4** (TGMR)**.** *For *$P^{k}\;(k\in N)$*, the state *$s_{i}\in S$* is considered TGMR stability iff for each state *$s_{j}\in R^{k,+}(s_{i})$* there exists at least one state *$s_{u}\in R^{N\backslash \{k\}}(s_{j})$* satisfying *$s_{i}{≿}_{k}s_{u}$* (* $pv_{i}^{k}\geq pv_{u}^{k}$*), where *N\{k}* denotes the set of all CCPs except *$P^{k}$.

**Definition 5** (TSMR)**.** *For *$P^{k}\;(k\in N)$*, the state *$s_{i}\in S$* is recognized as TSMR stability iff for each state *$s_{j}\in R^{k,+}(s_{i})$* there exists at least one state *$s_{u}\in R^{N\backslash \{k\}}(s_{j})$* satisfying *$s_{i}{≿}_{k}s_{u}$* (* $pv_{i}^{k}\geq pv_{u}^{k}$*), and for all states *$s_{v}\in R^{k}(s_{u})$*, *$s_{i}{≿}_{k}s_{v}$.

**Definition 6** (TSEQ)**.** *For *$P^{k}\;(k\in N)$*, the state *$s_{i}\in S$* is considered TSEQ stability iff for each state *$s_{j}\in R^{k,+}(s_{i})$* there exists at least one state *$s_{u}\in R^{N\backslash \{k\},+}(s_{j})$* satisfying *$s_{i}{≿}_{k}s_{u}$.

Note 1: Linear aggregation is adopted in this study because it offers a transparent and interpretable way to combine statement importance and state support, and is consistent with the score-based preference construction in option prioritization. It also facilitates the derivation of deterministic state preference scores for subsequent GMCR stability analysis.

## 5. Illustrative Application

This section presents an illustrative application of the proposed framework to a supply chain carbon reduction conflict. The case is used to show the implementation of the proposed analytical procedure under a specified conflict setting.

### 5.1. Background

Environmental sustainability has emerged as a critical imperative in global economic development. China’s Circular Economy Promotion Plan (2020) promotes resource recycling mechanisms aimed at achieving carbon peaking by 2030 and carbon neutrality by 2060. While supply chain decarbonization enhances corporate sustainability and low-carbon competitiveness, it also faces operational challenges under China’s dual-control policies on energy consumption and intensity. Provincial power rationing measures have forced manufacturers to reduce production by 50–70% through intermittent operation.

In this context, Chen et al. [10] developed a supply chain conflict case concerning carbon emission reduction. Upstream suppliers are making substantial investments in energy-saving and low-carbon technologies to comply with national decarbonization mandates. However, these efforts often lead to direct income losses and generate trade-offs between emissions reduction and profitability. When suppliers transfer carbon mitigation costs downstream through cost pass-through mechanisms, manufacturers face increased product prices that may erode consumer demand. As a result, manufacturers are reluctant to bear the burden of decarbonization costs. This calls for a mutually acceptable solution between upstream and downstream firms to ensure effective emissions reduction while maintaining supply chain viability. Building on this case, the present study adopts its background and data while reformulating the format of the assessment information to incorporate the flexible preference information provided by the conflicting parties.

### 5.2. Supply Chain Conflict Problem Formulation

In this conflict, there are mainly two CCPs: Supplier $P^{1}$ and Manufacturer $P^{2}$. Assume that the supplier $P^{1}$ consists of four individuals, denoted as $P^{1}=\{P_{1}^{1},\;P_{2}^{1},\;P_{3}^{1},\;P_{4}^{1}\}$, and the manufacturer $P^{2}$ likewise consists of four individuals, denoted as $P^{2}=\{P_{1}^{2},\;P_{2}^{2},\;P_{3}^{2},\;P_{4}^{2}\}$. $P^{1}$ has two available options, denoted as $O^{1}=\{o_{1}^{1},\;o_{2}^{1}\}$. Option $o_{1}^{1}$ (reduce carbon emissions) involves achieving carbon emission reductions through investments in technological innovation and environmentally sustainable products. Option $o_{2}^{1}$ (transfer the cost) refers to shifting part or all of the carbon reduction costs to $P^{2}$ by increasing product prices. Similarly, $P^{2}$ has two options, denoted as $O^{2}=\{o_{1}^{2},\;o_{2}^{2}\}$. Option $o_{1}^{2}$ (participation in carbon reduction) entails jointly investing with $P^{1}$ in the research and development of low-carbon products, which requires a substantial initial investment. Option $o_{2}^{2}$ (allocate the cost) implies that, in the early stage, $P^{2}$ does not engage in technology research and development but instead shares the carbon emission reduction costs by accepting higher product prices. Although Option $o_{2}^{2}$ avoids upfront investment, it results in cost sharing over subsequent stages in the long run. The CCPs and options are summarized in Table 2.

Each option for $P^{1}$ and $P^{2}$ is assigned a numerical label and can be either selected (Y) or unselected (N). Formally, this conflict comprises 2^4^ = 16 possible states. However, due to logical dependencies, some states can be merged. For instance, when $P^{1}$ does not undertake carbon emission reduction, the conflict is automatically terminated, and the choices of other strategies no longer affect the outcome. After merging states, 9 feasible states remain. The possible feasible states are presented in Table 2.

**Figure 2:**
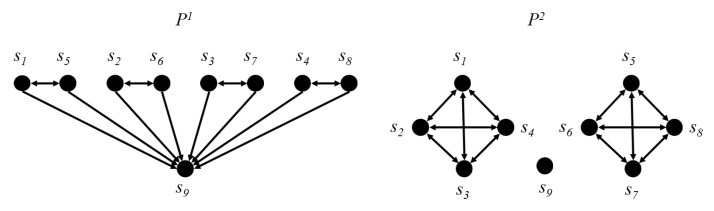
State transition diagrams.

The vertices of graph represent the states in the conflict. Directed arcs are used to represent each CCP’s unilateral transitions; the CCP associated with a given arc controls the one-step transition. An arc with two arrowheads indicates a reversible move. The state transition diagrams associated with $P^{1}$ and $P^{2}$ are presented in Figure 2.

Table 3 summarizes the statements associated with $P^{1}$ and $P^{2}$. The weight vectors of individuals in $P^{1}$ and $P^{2}$ are given as $\lambda^{1}=(0.25,\;0.25,\;0.25,\;0.25{)}^{T}$ and $\lambda^{2}={\left(0.25,\;0.25,\;0.25,\;0.25\right)}^{T}$, respectively. Specifically, the parameters α and β are assigned the values of 0.9 and 0.85, respectively.

Based on these statements, the pairwise comparison matrices over the statements provided by individuals $\left\{P_{1}^{1},\;P_{2}^{1},\;P_{3}^{1},\;P_{4}^{1}\right\}$ and $\left\{P_{1}^{2},\;P_{2}^{2},\;P_{3}^{2},\;P_{4}^{2}\right\}$ are denoted as $\left\{F^{1,1},\;F^{1,2},\;F^{1,3},\;F^{1,4}\right\}$ and $\left\{F^{2,1},\;F^{2,2},\;F^{2,3},\;F^{2,4}\right\}$, respectively. The specific pairwise comparison matrices are presented as follows:

$$F^{1,1}=\left[\begin{array}{cccc} - & 0.7 & 0.85 & 0.9\\ - & - & 0.8 & 0.85\\ - & - & - & 0.4\\ - & - & - & - \end{array}\right]\;F^{1,2}=\left[\begin{array}{cccc} - & 0.3 & 0.9 & 0.6\\ - & - & 0.9 & 0.65\\ - & - & - & 0.3\\ - & - & - & - \end{array}\right]\;F^{1,3}=\left[\begin{array}{cccc} - & 0.8 & 0.55 & 0.9\\ - & - & 0.3 & 0.6\\ - & - & - & 0.9\\ - & - & - & - \end{array}\right]\;F^{1,4}=\left[\begin{array}{cccc} - & 0.4 & 0.9 & 0.8\\ - & - & 0.75 & 0.9\\ - & - & - & 0.85\\ - & - & - & - \end{array}\right]\;F^{2,1}=\left[\begin{array}{ccc} - & 0.85 & 0.95\\ - & - & 0.9\\ - & - & - \end{array}\right]\;F^{2,2}=\left[\begin{array}{ccc} - & 0.3 & 0.85\\ - & - & 0.8\\ - & - & - \end{array}\right]\;F^{2,3}=\left[\begin{array}{ccc} - & 0.67 & 0.9\\ - & - & 0.5\\ - & - & - \end{array}\right]\;F^{2,4}=\left[\begin{array}{ccc} - & 0.9 & 0.6\\ - & - & 0.35\\ - & - & - \end{array}\right]$$

By applying Equation (2), the consistency levels of $\left\{F^{1,1},\;F^{1,2},\;F^{1,3},\;F^{1,4}\right\}$ and $\left\{F^{2,1},\;F^{2,2},\;F^{2,3},\;F^{2,4}\right\}$ are as follows: $CI(F^{1,1})=0.9$, $CI(F^{1,2})=0.917$, $CI(F^{1,3})=0.967$, $CI(F^{1,4})=0.85$, $CI(F^{2,1})=0.8$, $CI(F^{2,2})=0.833$, $CI(F^{2,3})=0.847$, $CI(F^{2,4})=0.9$. Using these weight vectors and Equation (4), the collective pairwise comparison matrices of $P^{1}$ and $P^{2}$ are derived as:

$$F^{1,c}=\left[\begin{array}{cccc} - & 0.55 & 0.8 & 0.8\\ - & - & 0.69 & 0.75\\ - & - & - & 0.61\\ - & - & - & - \end{array}\right]\;F^{2,c}=\left[\begin{array}{ccc} - & 0.68 & 0.83\\ - & - & 0.64\\ - & - & - \end{array}\right]$$

Based on the statements of each CCP, the initial flexible state assessment matrices provided by the individuals in $P^{1}$ and $P^{2}$ are presented as follows (Table 4 and Table 5):

Based on the weight vectors $\lambda^{1}$, $\lambda^{2}$, and Equation (12), the collective flexible state assessment matrices for CCPs are derived as follows (Table 6):

### 5.3. Consensus Reaching Process

#### 5.3.1. Consensus Reaching Process for Statement Importance

From Equation (5), the consensus levels of the pairwise comparison matrices of $P^{1}$ and $P^{2}$ are $CL(F^{1,1},\;\ldots ,\;F^{1,4})=0.832$ and $CL(F^{2,1},\;\ldots ,\;F^{2,4})=0.827$. Considering the consensus levels $CL(F^{1,1},\;\ldots ,\;F^{1,4})=0.832<\alpha$ and $CL(F^{2,1},\;\ldots ,\;F^{2,4})=0.827<\alpha$ are unacceptable, the minimum adjustment consensus model with fairness concern for statements is employed to derive the final consensual collective statement assessments within CCPs. The specific optimization model $M_{2}\prime$ for pairwise comparison matrices of $P^{1}$ is formulated below:

$$\begin{array}{l} \min(a_{12}^{1,1}+a_{13}^{1,1}+\ldots +a_{24}^{1,4}+a_{34}^{1,4})\\ s.t.\left\{\begin{array}{l} a_{12}^{1,1}\geq f_{12}^{1,1}-{\bar{f}}_{12}^{1,1},\;a_{12}^{1,1}\geq {\bar{f}}_{12}^{1,1}-f_{12}^{1,1},\;\ldots ,\;a_{34}^{1,4}\geq f_{34}^{1,4}-{\bar{f}}_{34}^{1,4},\;a_{34}^{1,4}\geq {\bar{f}}_{34}^{1,4}-f_{34}^{1,4}\\ {\bar{f}}_{12}^{1,c}=0.25\cdot ({\bar{f}}_{12}^{1,1}+{\bar{f}}_{12}^{1,2}+{\bar{f}}_{12}^{1,3}+{\bar{f}}_{12}^{1,4}),\;\ldots ,\;{\bar{f}}_{34}^{1,c}=0.25\cdot ({\bar{f}}_{34}^{1,1}+{\bar{f}}_{34}^{1,2}+{\bar{f}}_{34}^{1,3}+{\bar{f}}_{34}^{1,4})\\ 1-\frac{2}{4\times 4\times 3}\cdot (b_{12}^{1,1}+b_{13}^{1,1}+\ldots +b_{24}^{1,4}+b_{34}^{1,4})\geq 0.9\\ b_{12}^{1,1}\geq {\bar{f}}_{12}^{1,1}-{\bar{f}}_{12}^{1,c},\;b_{12}^{1,1}\geq {\bar{f}}_{12}^{1,c}-{\bar{f}}_{12}^{1,1},\;\ldots ,\;b_{34}^{1,4}\geq {\bar{f}}_{34}^{1,4}-{\bar{f}}_{34}^{1,c},\;b_{34}^{1,4}\geq {\bar{f}}_{34}^{1,c}-{\bar{f}}_{34}^{1,4}\\ 1-\frac{4}{4\times 3\times 2}\cdot (c_{123}^{1,1}+c_{134}^{1,1}+c_{124}^{1,1}+c_{234}^{1,1})\geq 0.85,\;\ldots ,\;1-\frac{4}{4\times 3\times 2}\cdot (c_{123}^{1,4}+c_{134}^{1,4}+c_{124}^{1,4}+c_{234}^{1,4})\geq 0.85\\ c_{123}^{1,1}\geq {\bar{f}}_{12}^{1,1}+{\bar{f}}_{23}^{1,1}-{\bar{f}}_{13}^{1,1}-0.5,\;c_{123}^{1,1}\geq -{\bar{f}}_{12}^{1,1}-{\bar{f}}_{23}^{1,1}+{\bar{f}}_{13}^{1,1}+0.5,\;\ldots ,\\ c_{234}^{1,4}\geq {\bar{f}}_{23}^{1,4}+{\bar{f}}_{34}^{1,4}-{\bar{f}}_{24}^{1,4}-0.5,\;c_{234}^{1,4}\geq -{\bar{f}}_{23}^{1,4}-{\bar{f}}_{34}^{1,4}+{\bar{f}}_{24}^{1,4}+0.5 \end{array}\right. \end{array}.$$

The solution obtained from the above model is then used as a fixed parameter, D=1.4 in model $M_{1}\prime$ for pairwise comparison matrices of $P^{1}$ in the subsequent model to represent the minimum adjustment required to achieve a consensual collective assessment. The specific optimization model $M_{1}\prime$ for pairwise comparison matrices of $P^{1}$ is as follows:

$$\begin{array}{l} \min\varepsilon \\ s.t.\left\{\begin{array}{l} {\bar{f}}_{12}^{1,c}=0.25\cdot ({\bar{f}}_{12}^{1,1}+{\bar{f}}_{12}^{1,2}+{\bar{f}}_{12}^{1,3}+{\bar{f}}_{12}^{1,4}),\;\ldots ,\;{\bar{f}}_{34}^{1,c}=0.25\cdot ({\bar{f}}_{34}^{1,1}+{\bar{f}}_{34}^{1,2}+{\bar{f}}_{34}^{1,3}+{\bar{f}}_{34}^{1,4})\\ \frac{1}{6}\cdot [(a_{12}^{1,1}+\ldots +a_{34}^{1,1})-\frac{1}{3}(a_{12}^{1,2}+\ldots +a_{34}^{1,4})]\leq \varepsilon ,\;\ldots ,\;\frac{1}{6}\cdot [(a_{12}^{1,2}+\ldots +a_{34}^{1,4})-\frac{1}{3}(a_{12}^{1,2}+\ldots +a_{34}^{1,3})]\leq \varepsilon \\ a_{12}^{1,1}\geq f_{12}^{1,1}-{\bar{f}}_{12}^{1,1},\;a_{12}^{1,1}\geq {\bar{f}}_{12}^{1,1}-f_{12}^{1,1},\;\ldots ,\;a_{34}^{1,4}\geq f_{34}^{1,4}-{\bar{f}}_{34}^{1,4},\;a_{34}^{1,4}\geq {\bar{f}}_{34}^{1,4}-f_{34}^{1,4}\\ 1-\frac{2}{4\times 4\times 3}\cdot (b_{12}^{1,1}+\ldots +b_{34}^{1,4})\geq 0.9\\ b_{12}^{1,1}\geq {\bar{f}}_{12}^{1,1}-{\bar{f}}_{12}^{1,c},\;b_{12}^{1,1}\geq {\bar{f}}_{12}^{1,c}-{\bar{f}}_{12}^{1,1},\;\ldots ,\;b_{34}^{1,4}\geq {\bar{f}}_{34}^{1,4}-{\bar{f}}_{34}^{1,c},\;b_{34}^{1,4}\geq {\bar{f}}_{34}^{1,c}-{\bar{f}}_{34}^{1,4}\\ 1-\frac{4}{4\times 3\times 2}\cdot (c_{123}^{1,1}+c_{134}^{1,1}+c_{124}^{1,1}+c_{234}^{1,1})\geq 0.85,\;\ldots ,\;1-\frac{4}{4\times 3\times 2}\cdot (c_{123}^{1,4}+c_{134}^{1,4}+c_{124}^{1,4}+c_{234}^{1,4})\geq 0.85\\ c_{123}^{1,1}\geq {\bar{f}}_{12}^{1,1}+{\bar{f}}_{23}^{1,1}-{\bar{f}}_{13}^{1,1}-0.5,\;c_{123}^{1,1}\geq -{\bar{f}}_{12}^{1,1}-{\bar{f}}_{23}^{1,1}+{\bar{f}}_{13}^{1,1}+0.5,\;\ldots ,\\ c_{234}^{1,4}\geq {\bar{f}}_{23}^{1,4}+{\bar{f}}_{34}^{1,4}-{\bar{f}}_{24}^{1,4}-0.5,\;c_{234}^{1,4}\geq -{\bar{f}}_{23}^{1,4}-{\bar{f}}_{34}^{1,4}+{\bar{f}}_{24}^{1,4}+0.5\\ a_{12}^{1,1}+\ldots +a_{34}^{1,4}=1.4 \end{array}\right. \end{array}.$$

After solving the aforementioned model, the optimized value of ε and the adjusted consensual collective statement assessments for $P^{1}$ are obtained. The same process is repeated to derive the results for $P^{2}$. Specifically, the minimum adjustment values are found to be D=1.4 for $P^{1}$ and D=0.83 for $P^{2}$. Based on these adjustment levels, the corresponding ε=0.022 for $P^{1}$ and ε=0 for $P^{2}$, reflecting the acceptable levels of perceived fairness during the CRP. The adjusted consensual collective statement assessments for $P^{1}$ and $P^{2}$ are summarized as:

$${\bar{F}}^{1,c}=\left[\begin{array}{cccc} - & 0.596 & 0.85 & 0.821\\ - & - & 0.75 & 0.7\\ - & - & - & 0.533\\ - & - & - & - \end{array}\right]\;{\bar{F}}^{2,c}=\left[\begin{array}{ccc} - & 0.67 & 0.85\\ - & - & 0.678\\ - & - & - \end{array}\right]$$

#### 5.3.2. Consensus Reaching Process for State Support

The minimum adjustment consensus model with fairness concern for states is used to obtain final consensual collective state assessments within CCPs. Specific models for $P^{1}$ and $P^{2}$ are formulated based on model $M_{3}\prime$ and model $M_{4}\prime$. The specific optimization model $M_{4}\prime$ for pairwise comparison matrices of $P^{1}$ is formulated below:

$$\begin{array}{l} \min(a_{11}^{1,1}+a_{12}^{1,1}+\ldots +a_{43}^{1,4}+a_{44}^{1,4})\\ s.t.\left\{\begin{array}{l} a_{11}^{1,1}\geq v_{11}^{1,1}-{\bar{v}}_{11}^{1,1},\;a_{11}^{1,1}\geq {\bar{v}}_{11}^{1,1}-v_{11}^{1,1},\;\ldots ,\;a_{44}^{1,4}\geq v_{44}^{1,4}-{\bar{v}}_{44}^{1,4},\;a_{44}^{1,4}\geq {\bar{v}}_{44}^{1,4}-v_{44}^{1,4}\\ {\bar{v}}_{11}^{1,c}=0.25\cdot ({\bar{v}}_{11}^{1,1}+{\bar{v}}_{11}^{1,2}+{\bar{v}}_{11}^{1,3}+{\bar{v}}_{11}^{1,4}),\;\ldots ,\;{\bar{v}}_{44}^{1,c}=0.25\cdot ({\bar{v}}_{44}^{1,1}+{\bar{v}}_{44}^{1,2}+{\bar{v}}_{44}^{1,3}+{\bar{v}}_{44}^{1,4})\\ 1-\frac{1}{4\times 9\times 4}\cdot (b_{11}^{1,1}+b_{12}^{1,1}+\ldots +b_{43}^{1,4}+b_{44}^{1,4})\geq 0.9\\ b_{11}^{1,1}\geq {\bar{v}}_{11}^{1,1}-{\bar{v}}_{11}^{1,c},\;b_{11}^{1,1}\geq {\bar{v}}_{11}^{1,c}-{\bar{v}}_{11}^{1,1},\;\ldots ,\;b_{44}^{1,4}\geq {\bar{v}}_{44}^{1,4}-{\bar{v}}_{44}^{1,c},\;b_{44}^{1,4}\leq {\bar{v}}_{44}^{1,4}-{\bar{v}}_{44}^{1,c} \end{array}\right. \end{array}.$$

The solution obtained from the above model is then used as a fixed parameter, D=4.92 in model $M_{3}\prime$ for the statement assessments of $P^{1}$ in the subsequent model to represent the minimum adjustment required to achieve a consensual collective assessment. The specific optimization model $M_{3}\prime$ for pairwise comparison matrices of $P^{1}$ as follows:

$$\begin{array}{l} \min\varepsilon \\ s.t.\left\{\begin{array}{l} {\bar{v}}_{11}^{1,c}=0.25\cdot ({\bar{v}}_{11}^{1,1}+{\bar{v}}_{11}^{1,2}+{\bar{v}}_{11}^{1,3}+{\bar{v}}_{11}^{1,4}),\;\ldots ,\;{\bar{v}}_{44}^{1,c}=0.25\cdot ({\bar{v}}_{44}^{1,1}+{\bar{v}}_{44}^{1,2}+{\bar{v}}_{44}^{1,3}+{\bar{v}}_{44}^{1,4})\\ 3\times (a_{11}^{1,1}+\ldots +a_{44}^{1,1})-(a_{11}^{1,2}+\ldots +a_{44}^{1,4})\leq \varepsilon ,\;\ldots ,\;3\times (a_{11}^{1,4}+\ldots +a_{44}^{1,4})-(a_{11}^{1,1}+\ldots +a_{44}^{1,3})\leq \varepsilon \\ a_{11}^{1,1}\geq v_{11}^{1,1}-{\bar{v}}_{11}^{1,1},\;a_{11}^{1,1}\geq {\bar{v}}_{11}^{1,1}-v_{11}^{1,1},\;\ldots ,\;a_{44}^{1,4}\geq v_{44}^{1,4}-{\bar{v}}_{44}^{1,4},\;a_{44}^{1,4}\geq {\bar{v}}_{44}^{1,4}-v_{44}^{1,4}\\ 1-\frac{1}{4\times 9\times 4}\cdot (b_{11}^{1,1}+b_{12}^{1,1}+\ldots +b_{43}^{1,4}+b_{44}^{1,4})\geq 0.9\\ b_{11}^{1,1}\geq {\bar{v}}_{11}^{1,1}-{\bar{v}}_{11}^{1,c},\;b_{11}^{1,1}\geq {\bar{v}}_{11}^{1,c}-{\bar{v}}_{11}^{1,1},\;\ldots ,\;b_{44}^{1,4}\geq {\bar{v}}_{44}^{1,4}-{\bar{v}}_{44}^{1,c},\;b_{44}^{1,4}\leq {\bar{v}}_{44}^{1,4}-{\bar{v}}_{44}^{1,c}\\ a_{11}^{1,1}+a_{12}^{1,1}+\ldots +a_{43}^{1,4}+a_{44}^{1,4}=4.92 \end{array}\right. \end{array}$$

Upon solving the aforementioned models, the minimum adjustments are D=4.92 for $P^{1}$ and D=2.95 for $P^{2}$, indicating moderate preference modifications for both parties. Based on these adjustment levels, the corresponding ε=0 for $P^{1}$ and ε=0.003 for $P^{2}$. The resulting consensual collective state assessments after adjustment are presented in Table 7.

### 5.4. Stability Analysis Based on Preference Threshold

The statement weights can be computed using Equation (4). Accordingly, the resulting weight vectors are $\omega^{1}={\left(0.3778,\;0.3090,\;0.1556,\;0.1576\right)}^{T}$ and $\omega^{2}={\left(0.5067,\;0.3358,\;0.1575\right)}^{T}$. Based on the weight vectors and the adjusted collective assessment matrices ${\bar{V}}^{1,c}$ and ${\bar{V}}^{2,c}$, the preference scores for each state can be computed using Equation (5). Then the preference vectors are derived as $PV^{1}=(0.718,\;0.493,\;0.816,\;0.494,\;0.577,\;0.534,\;0.692,\;0.572,\;0.491{)}^{T}$ and $PV^{2}=(0.269,\;0.486,\;0.481,\;0.411,\;0.159,\;0.412,\;0.397,\;0.394,\;0.591{)}^{T}$. Consequently, the preferences of $P^{1}$ and $P^{2}$ regarding feasible states are as follows: $s_{3}≻s_{1}≻s_{7}≻s_{5}≻s_{8}≻s_{6}≻s_{4}≻s_{2}≻s_{9}$ for $P^{1}$ and $s_{9}≻s_{2}≻s_{3}≻s_{6}≻s_{4}≻s_{7}≻s_{8}≻s_{1}≻s_{5}$ for $P^{2}$.

It is assumed that both $P^{1}$ and $P^{2}$ adopt a preference threshold value of 0.05. The CCPs may adjust these values as needed to reflect specific circumstances. Once the preference thresholds have been established, the stability of each state under these thresholds can be assessed in accordance with Definitions 3–6, as summarized in Table 8. Among these states, $s_{2}$, $s_{3}$, $s_{6}$, $s_{8}$, and $s_{9}$ constitute equilibria under all four stability definitions.

Among these equilibrium states, state $s_{2}$ represents a low-cost passive cooperation equilibrium: the supplier reduces carbon emissions without transferring costs, while the manufacturer does not participate in carbon reduction but is willing to allocate the cost. State $s_{3}$ describes a joint-investment equilibrium: the supplier reduces emissions without cost transfer, and the manufacturer participates in carbon reduction without allocating the cost. State $s_{6}$ denotes a cost-externalization equilibrium, where the supplier reduces emissions and transfers the cost to the manufacturer, and the manufacturer is also willing to allocate the cost without participating in carbon reduction. State $s_{8}$ shows a joint-investment and cost pass-through equilibrium: the supplier reduces emissions and transfers the cost, while the manufacturer both participates in carbon reduction and allocates the cost. State $s_{9}$ represents a do-nothing equilibrium: the supplier takes no emission reduction action, leading to automatic conflict termination with no low-carbon efforts from either party.

## 6. Conclusions

This study develops an integrated framework that incorporates consensus-based GDM into the GMCR framework to address complex conflict problems characterized by inter-entity conflicts and intra-entity assessment divergences. By extending traditional option prioritization methods, the proposed approach introduces flexible assessments, employing pairwise comparisons to quantify the importance of statements and continuous numerical values within the interval [0, 1] to capture nuanced state support, thereby overcoming the limitations of ordinal rankings and binary assessments. Furthermore, two minimum adjustment consensus models incorporating fairness concerns are developed to reconcile divergent individual assessments within each CCP. These models not only minimize deviations from original assessments but also embed fairness concerns to enhance perceived equity in the CRP. Based on the adjusted assessments, consensus-based preferences among all CCPs are derived and subsequently integrated into the GMCR stability analysis to identify equilibrium solutions. The proposed framework is applied to a supply chain carbon emission reduction conflict.

Meanwhile, three interesting and noteworthy directions for future research are outlined below: (1) Empirical application and robustness validation should be strengthened by applying the framework to real-world conflicts, acquiring empirical data through expert elicitation and stakeholder validation, and conducting systematic robustness testing. (2) Heterogeneous decision makers and behavioral extensions deserve further investigation, as individuals may differ in expertise, authority, influence, bargaining power, trust relationships, and strategic incentives. Incorporating these factors into individual weights and fairness measures can better capture the multidimensional nature of fairness concern. (3) Methodological enhancement is needed to examine the trade-offs among fairness, adjustment minimization, consensus, and consistency, and to capture potential nonlinear interdependencies between statement weights and state support values through interaction terms, Choquet integrals, or network-based aggregation mechanisms.

## Figures and Tables

**Table 1:** Comparison analysis of related GMCR studies.

Reference	Preference Structure	Whether to Use Option Prioritization	Whether to Consider CCPs	Whether to Consider Consensus Within the CCP	Whether to Consider Fairness Consideration
Fang et al. (2003) [19]	Crisp preference	Yes	No	No	No
Hamouda et al. (2004) [23]	Strength of preference	No	No	No	No
Rêgo et al. (2021) [27]	Probabilistic preference	No	No	No	No
Wu et al. (2021) [20]	Hesitant fuzzy preference	Yes	Yes	No	No
Wu et al. (2023) [13]	Hesitant fuzzy preference	No	Yes	No	No
Wang et al. (2023) [25]	Intuitionistic preference	No	No	No	No
Chen et al. (2023) [10]	Hesitant fuzzy preference	Yes	No	No	No
Liu et al. (2024) [14]	Linguistic preference	No	No	No	No
Zhang et al. (2024) [24]	Grey preference	No	No	No	No
Li et al. (2024) [21]	Grey preference	Yes	No	No	No
Yu et al. (2025) [22]	Unknown preference	Yes	No	No	No
This study	Pairwise comparison relation	Yes	Yes	Yes	Yes

**Table 2:** Feasible states of $P^{1}$ and $P^{2}$.

CCPs	Options	Feasible States								
		$s_{1}$	$s_{2}$	$s_{3}$	$s_{4}$	$s_{5}$	$s_{6}$	$s_{7}$	$s_{8}$	$s_{9}$
$P^{1}$	Reduce carbon emissions $\left(o_{1}^{1}\right)$	Y	Y	Y	Y	Y	Y	Y	Y	N
	Transfer the cost $\left(o_{2}^{1}\right)$	N	N	N	N	Y	Y	Y	Y	-
$P^{2}$	Participate in carbon reduction $\left(o_{1}^{2}\right)$	N	N	Y	Y	N	N	Y	Y	-
	Allocate the cost $\left(o_{2}^{2}\right)$	N	Y	N	Y	N	Y	N	Y	-

**Table 3:** Statements associated with $P^{1}$ and $P^{2}$ in the conflict scenario.

CCPs	Statements	Explanation
$P^{1}$	$o_{1}^{1}$	$P^{1}$ wants to reduce the carbon emissions.
	−$o_{2}^{2}$	$P^{1}$ wants $P^{2}$ not to take the allocate the cost option.
	$o_{1}^{2}$	$P^{1}$ wants $P^{2}$ to co-invest in low-carbon new product research and development with $P^{1}$.
	$o_{2}^{1}$ iff $o_{2}^{2}$	$P^{1}$ would like to transfer cost iff $P^{2}$ chooses to allocate cost.
$P^{2}$	−$o_{1}^{1}$	$P^{2}$ does not want $P^{1}$ to reduce the carbon emissions.
	$o_{1}^{2}$|$o_{2}^{2}$	$P^{2}$ is not sure whether to co-invest in low-carbon or allocate cost.
	−$o_{2}^{1}$	$P^{2}$ does not like $P^{1}$ to transfer the cost.

**Table 4:** Flexible state assessment matrices for $P^{1}$.

	$V^{1,1}$				$V^{1,2}$				$V^{1,3}$				$V^{1,4}$			
	$\Omega_{1}^{1}$	$\Omega_{2}^{1}$	$\Omega_{3}^{1}$	$\Omega_{4}^{1}$	$\Omega_{1}^{1}$	$\Omega_{2}^{1}$	$\Omega_{3}^{1}$	$\Omega_{4}^{1}$	$\Omega_{1}^{1}$	$\Omega_{2}^{1}$	$\Omega_{3}^{1}$	$\Omega_{4}^{1}$	$\Omega_{1}^{1}$	$\Omega_{2}^{1}$	$\Omega_{3}^{1}$	$\Omega_{4}^{1}$
$s_{1}$	0.9	0.9	0.1	0.95	0.6	0.8	0.4	0.6	0.8	0.8	0.1	0.95	0.95	0.6	0.5	0.8
$s_{2}$	0.9	0.1	0.15	0.1	0.6	0.1	0.5	0.05	0.8	0.5	0.1	0.5	0.95	0.5	0.5	0.4
$s_{3}$	0.9	0.95	0.7	0.9	0.9	0.7	0.8	0.7	0.9	0.65	0.9	0.9	0.6	0.95	0.6	0.6
$s_{4}$	0.9	0.4	0.9	0.2	0.95	0.1	0.7	0.1	0.65	0.1	0.4	0.4	0.6	0.5	0.9	0.5
$s_{5}$	0.9	0.8	0.3	0.1	0.7	0.9	0.1	0.5	0.6	0.6	0.1	0.1	0.9	0.9	0.1	0.2
$s_{6}$	0.9	0.45	0.2	0.95	0.75	0.1	0.45	0.95	0.87	0.5	0.7	0.7	0.7	0.1	0.3	0.6
$s_{7}$	0.9	0.9	0.6	0.1	0.9	0.8	0.9	0.1	0.7	0.6	0.45	0.45	0.75	0.9	0.7	0.4
$s_{8}$	0.9	0.1	0.9	0.9	0.8	0.1	0.7	0.8	0.6	0.5	0.6	0.6	0.6	0.4	0.95	0.6
$s_{9}$	0.1	0.8	0.2	0.95	0.1	0.8	0.05	0.7	0.45	0.9	0.6	0.6	0.5	0.5	0.1	0.9

**Table 5:** Flexible state assessment matrices for $P^{2}$.

	$V^{2,1}$			$V^{2,2}$			$V^{2,3}$			$V^{2,4}$		
	$\Omega_{1}^{2}$	$\Omega_{2}^{2}$	$\Omega_{3}^{2}$	$\Omega_{1}^{2}$	$\Omega_{2}^{2}$	$\Omega_{3}^{2}$	$\Omega_{1}^{2}$	$\Omega_{2}^{2}$	$\Omega_{3}^{2}$	$\Omega_{1}^{2}$	$\Omega_{2}^{2}$	$\Omega_{3}^{2}$
$s_{1}$	0.05	0.1	0.95	0.5	0.2	0.8	0.1	0.1	0.9	0.2	0.5	0.6
$s_{2}$	0.2	0.95	0.95	0.1	0.7	0.5	0.4	0.9	0.7	0.1	0.6	0.9
$s_{3}$	0.1	0.9	0.9	0.2	0.8	0.9	0.5	0.6	0.6	0.1	0.9	0.8
$s_{4}$	0.1	0.95	0.9	0.45	0.6	0.6	0.05	0.6	0.8	0.05	0.95	0.9
$s_{5}$	0.1	0.1	0.1	0.1	0.1	0.15	0.2	0.1	0.05	0.5	0.5	0.5
$s_{6}$	0.1	0.95	0.1	0.4	0.6	0.3	0.3	0.9	0.1	0.05	0.7	0.4
$s_{7}$	0.1	0.95	0.1	0.5	0.8	0.5	0.1	0.6	0.1	0.2	0.7	0.3
$s_{8}$	0.1	0.9	0.1	0.1	0.6	0.45	0.5	0.7	0.5	0.2	0.9	0.1
$s_{9}$	0.9	0.1	0.9	0.8	0.2	0.95	0.6	0.5	0.6	0.9	0.05	0.7

**Table 6:** Collective flexible state assessment matrices for $P^{1}$ and $P^{2}$.

	$V^{1,c}$				$V^{2,c}$		
	$\Omega_{1}^{1}$	$\Omega_{2}^{1}$	$\Omega_{3}^{1}$	$\Omega_{4}^{1}$	$\Omega_{1}^{2}$	$\Omega_{2}^{2}$	$\Omega_{3}^{2}$
$s_{1}$	0.813	0.775	0.275	0.825	0.213	0.225	0.813
$s_{2}$	0.813	0.3	0.313	0.263	0.2	0.788	0.763
$s_{3}$	0.825	0.813	0.75	0.775	0.225	0.8	0.8
$s_{4}$	0.775	0.275	0.775	0.3	0.163	0.775	0.8
$s_{5}$	0.775	0.8	0.238	0.225	0.225	0.2	0.2
$s_{6}$	0.805	0.288	0.263	0.8	0.213	0.788	0.225
$s_{7}$	0.813	0.8	0.775	0.263	0.225	0.763	0.25
$s_{8}$	0.725	0.275	0.788	0.725	0.225	0.775	0.288
$s_{9}$	0.288	0.75	0.213	0.788	0.8	0.213	0.788

**Table 7:** Adjusted collective state assessment matrices for $P^{1}$ and $P^{2}$.

	$V^{1,c}$				$V^{2,c}$		
	$\Omega_{1}^{1}$	$\Omega_{2}^{1}$	$\Omega_{3}^{1}$	$\Omega_{4}^{1}$	$\Omega_{1}^{2}$	$\Omega_{2}^{2}$	$\Omega_{3}^{2}$
$s_{1}$	0.8	0.8	0.275	0.8	0.15	0.2	0.8
$s_{2}$	0.8	0.3	0.294	0.333	0.2	0.788	0.763
$s_{3}$	0.9	0.767	0.75	0.775	0.171	0.8	0.8
$s_{4}$	0.717	0.233	0.733	0.233	0.088	0.717	0.8
$s_{5}$	0.775	0.8	0.1	0.133	0.2	0.1	0.15
$s_{6}$	0.805	0.234	0.2	0.8	0.213	0.788	0.25
$s_{7}$	0.813	0.8	0.775	0.112	0.2	0.763	0.25
$s_{8}$	0.725	0.2	0.788	0.725	0.2	0.771	0.217
$s_{9}$	0.233	0.8	0.2	0.788	0.8	0.2	0.75

**Table 8:** Threshold stability analysis.

States	TNash			TGMR			TSMR			TSEQ		
	$P^{1}$	$P^{2}$	E	$P^{1}$	$P^{2}$	E	$P^{1}$	$P^{2}$	E	$P^{1}$	$P^{2}$	E
$s_{1}$	√			√			√			√		
$s_{2}$	√	√	*	√	√	*	√	√	*	√	√	*
$s_{3}$	√	√	*	√	√	*	√	√	*	√	√	*
$s_{4}$												
$s_{5}$				√			√			√		
$s_{6}$	√	√	*	√	√	*	√	√	*	√	√	*
$s_{7}$		√			√			√			√	
$s_{8}$	√	√	*	√	√	*	√	√	*	√	√	*
$s_{9}$	√	√	*	√	√	*	√	√	*	√	√	*

“√” denotes stability for a CCP under a specified stability definition, while “*” denotes stability for all CCPs, indicating an equilibrium state.

## Data Availability

The original contributions presented in this study are included in the article. Further inquiries can be directed to the corresponding author.

## Abbreviations

**Notions**	**Meanings**
GDM	Group Decision Making
CCP	Composite Conflicting Party
GMCR	Graph Model for Conflict Resolution
CRP	Consensus Reaching Process
Nash	Nash Stability
GMR	General Metarationality Stability
SMR	Symmetric Metarationality Stability
SEQ	Sequential Stability
iff	If and Only If
TNash	Threshold-based Nash Stability
TGMR	Threshold-based General Metarationality Stability
TSMR	Threshold-based Symmetric Metarationality Stability
TSEQ	Threshold-based Sequential Stability

## Notations

**Symbol**	**Description**
$P=\{P^{1},\;\ldots ,P^{k},\;\ldots ,P^{n}\}$	Set of n(n≥2) CCPs
$P^{k}=\{P_{1}^{k},\;\ldots ,P_{l}^{k},\;\ldots ,P_{m(k)}^{k}\}$	Set of individuals within $P^{k}$
$\lambda^{k}={\left(\lambda_{1}^{k},\;\lambda_{2}^{k},\;\ldots ,\;\lambda_{m(k)}^{k}\right)}^{T}$	Weight vector of the individuals within $P^{k}$
$O^{k}=\{o_{1}^{k},\;o_{2}^{k},\;\ldots ,\;o_{h^{k}}^{k}\}$	Set of options associated with CCP $P^{k}$
$S=\{s_{1},\;s_{2},\;\ldots ,\;s_{h}\}$	Set of feasible states
$A^{k}\subseteq S\times S$	Oriented arcs provided by $P^{k}$
$\Omega^{k}=\{\Omega_{1}^{k},\;\Omega_{2}^{k},\;\ldots ,\;\Omega_{g(k)}^{k}\}$	Set of statements associated with $P^{k}$
$PV^{k}={\left(pv_{1}^{k},\;pv_{2}^{k},\;\ldots ,\;pv_{h}^{k}\right)}^{T}$	Preference vector over states for $P^{k}$
$F^{k,l}={\left(f_{ij}^{k,l}\right)}_{g(k)\times g(k)}$	The pairwise comparison matrix over statements of $P_{l}^{k}$
$F^{k,c}={\left(f_{ij}^{k,c}\right)}_{g(k)\times g(k)}$	The collective pairwise comparison matrix over statements for $P^{k}$
${\bar{F}}^{k,l}={\left({\bar{f}}_{ij}^{k,l}\right)}_{g(k)\times g(k)}$	The adjusted pairwise comparison matrix over statements of $P_{l}^{k}$
${\bar{F}}^{k,c}={\left({\bar{f}}_{ij}^{k,c}\right)}_{g(k)\times g(k)}$	The adjusted collective pairwise comparison matrix over statements for $P^{k}$
$\omega^{k}={\left(\omega_{1}^{k},\;\omega_{2}^{k},\;\ldots ,\;\omega_{g(k)}^{k}\right)}^{T}$	Priority vector of statements for $P^{k}$
$V^{k,l}={\left(v_{ij}^{k,l}\right)}_{h\times g(k)}$	The flexible state assessments matrix of $P_{l}^{k}$
$V^{k,c}={\left(v_{ij}^{k,c}\right)}_{h\times g(k)}$	The collective flexible state assessments matrix for $P^{k}$
${\bar{V}}^{k,l}={\left({\bar{v}}_{ij}^{k,l}\right)}_{h\times g(k)}$	The adjusted flexible state assessments matrix of $P^{k}$
${\bar{V}}^{k,c}={\left({\bar{v}}_{ij}^{k,c}\right)}_{h\times g(k)}$	The adjusted flexible state assessments matrix for $P^{k}$
α	The consensus threshold
β	The consistency threshold
ε	The fairness threshold
$\gamma^{k}$	The preference threshold of $P^{k}$
